# A Comparative Study of Client-Level Stabilization Techniques in Federated Learning for Robust Plant Disease Recognition

**DOI:** 10.3390/s26206566

**Published:** 2026-10-16

**Authors:** Ana-Maria Cristea, Ciprian Dobre

**Affiliations:** 1 Faculty of Automatic Control and Computer Science, National University of Science and Technology POLITEHNICA Bucharest, 060042 Bucharest, Romania; ciprian.dobre@upb.ro; 2 National Institute for Research and Development in Informatics, 011455 Bucharest, Romania; 3 Romanian Academy of Scientists, 030167 Bucharest, Romania

**Keywords:** federated learning, client specialization, data heterogeneity, plant disease, convolutional neural networks, field conditions

## Abstract

The successful integration of artificial intelligence (AI) algorithms in precision agriculture provided farmers with the means to identify potential issues, such as early disease detection or pest infestations, by making crop-related predictions. The new technological developments in this field improve the functioning of traditional agriculture by reducing the dependence on specialized human expertise and facilitating greater access to diagnostic procedures. However, most studies conducted in this area rely on centralized training of AI models, which leads to increased costs and raised privacy-related concerns. Federated learning (FL) offers a compelling framework for collaboratively training machine learning (ML) models on distributed datasets without centralizing the data owned by each participant. FL offers significant advantages in the preservation of data privacy, facilitates the collaborative exploitation of geographically distributed data sources, and reduces the risks associated with centralized collection. In this paper, we investigate the current limitations and performance boundaries of existing Federated and privacy-preserving learning in distributed sensing systems for plant disease detection, in scenarios that closely resemble real-world conditions. Our analysis specifically examined how FL aggregation algorithms, such as FedAvg, FedAvgM and FedProx, work in a custom multi-client experimental setup where data is partitioned so that each participant observes only those symptom patterns associated with specific environmental and climatic conditions. We propose further partial aggregation of the local model, an approach where we send only the model backbone to the central server, while the classification head of each participant remains strictly on the client side. The construction of environmentally heterogeneity is combined with a globally representative stabilizer node designed to preserve knowledge across disease classes that may be absent from certain clients.

## 1. Introduction and Background

Ensuring the integrity of annual crop production is a fundamental component of sustainable agricultural systems, having a direct impact on population well-being. Stable and quality harvests are essential to ensure the constant access of communities to food, influencing the level of prices, and reducing the risk of food insecurity or famine in certain regions. Early and accurate detection of plant pathologies is essential in mitigating economic damage and allowing timely intervention. At a global scale, pathogens and pests cause significant yields loss in food crops [1], and the economic impact is considerable even for a single crop. According to Bandara et al. [2], diseases of soybean alone were estimated to have caused losses of approximately 95.5 billion dollars in the United States over two decades. Climate change is expected to aggravate this situation, as shifts in temperature, humidity and precipitation alter the distribution and severity of plant disease and increase the pressure on farmers to adapt their practices [3,4]. Al-Kilani et al. [5] consider that such adaptations are often limited by the farmers access to resources, climatic data and research output, which highlights the need for tools that bring timely and reliable diagnostic support. Preventing disease development at an early stage can limit disease spread, reduce the need for expensive pesticide application, and support efficient use of resources.

In recent years, deep learning has emerged as the leading approach for plant disease identification, with convolutional neural networks (CNNs) and, more recently, vision transformer (ViT)-based and hybrid CNN–ViT architectures achieving classification accuracies exceeding 98% on benchmark datasets such as PlantVillage [6]. However, these results have been obtained largely under a centralized training paradigm, in which large volumes of labeled images are aggregated and stored in a single repository before model training. Farmers and agricultural stakeholders have shown a persistent reluctance to share raw farm data due to unresolved questions of data ownership, liability, and the absence of standardized governance frameworks regulating how such data can be collected, stored, and reused by third parties [7]. These concerns are compounded by practical infrastructure limitations, as many rural and field-based settings still lack the reliable high-bandwidth connectivity required to transmit large volumes of raw imagery to centralized cloud servers [8,9]. Taken together, data fragmentation, unclear ownership rights, privacy risks, and limited connectivity constitute structural barriers that restrict direct transfer of raw images to a central server, motivating the need for training paradigms such as FL, that allow model development to occur without requiring raw data to leave its original source [10,11].

Federated learning (FL), first introduced by McMahan et al. [12] as a decentralized paradigm for training shared models without centralizing data, provides an attractive alternative to solve the problems of traditional plant detection systems. FL enables multiple clients, such as farms, laboratories, or greenhouse monitoring stations, to collaboratively train a global machine learning (ML) model without sharing raw images. Each participant in the training process updates its model locally and sends only the learned parameters to a central server for aggregation. Beyond preserving data privacy, this paradigm naturally aligns with the distributed nature of agricultural environments, where collecting large labeled datasets in a single location is often infeasible due to variations in climate conditions, temperature regimes, pest prevalence, soil properties, and other local conditions. These factors reinforce a longstanding challenge of traditional FL algorithms, which typically experience substantial degradation when exposed to non-IID data. This heterogeneity intensifies client drift during training, leading to unstable convergence and reduced generalization.

Recent studies have started to integrate FL into plant disease recognition systems, exploring settings that resemble multi-client privacy preserving design. Mamba Kabala et al. [13] investigate which deep-learning architectures and FL configurations yield the best performance for crop disease classification, focusing mainly on the number of clients, communication rounds, local iterations and data quality. However, the dataset is equally and randomly partitioned across clients, therefore, the experimental setup does consider a non-independent and identically distributed (non-IID) scenario. Furthermore, federated training relies exclusively on FedAvg [12] model aggregation and does not evaluate alternative strategies. Aggarwal et al. [14] proposed a federated transfer learning (FTL) framework for rice-leaf disease classification, evaluating EfficientNetB3 and MobileNetV2 across four clients and considering IID and non-IID distributions. Although [14] explicitly considers non-IID data, it does not provide an in-depth investigation of the impact of heterogeneity-related constraints on FL performances. Nevertheless, both [13,14] rely exclusively on FedAvg, which is known to perform effectively in relatively homogeneous or IID distributions. Unlike previous studies, Caminha et al. [15] go further in explicitly modeling heterogeneous client data among five federated clients. The authors did not propose a novel heterogeneous aware aggregation algorithm and choose the classical FedAvg formulation. Neither [14] nor [15] provide a systematic analysis of varying degrees of statistical heterogeneity or compare aggregation strategies specifically designed to mitigate client drift.

In contrast to previous approaches, Chorney et al. [16] focus solely on a highly heterogeneous multi-client environment, marked by substantial differences in the dataset structure. The authors simulated five farms using five different real datasets, each different in terms of size, class distribution, class imbalance, and image characteristics. They used FedAvg as the starting point for aggregation, but also evaluated several strategies and introduced two hybrid pretraining approaches. Chorney et al. [16] concluded that large clients can dominate global optimization and affect small clients with substantially different local distributions.

Although existing studies have progressively considered heterogeneous multi-site agricultural data or non-IID distributions, most either rely on the classic FedAvg algorithm for aggregation or employ statistically simulated partitions (e.g., Dirichlet-based label-skew sampling) that reproduce heterogeneity as a synthetic artifact, decoupled from any real agronomic or climatic rationale. To the best of our knowledge, no prior work has explicitly grounded the federated partitioning of plant disease data in the environmental and agronomic conditions that causally drive disease-distribution shift across sites, treating heterogeneity as an artifact of the data-generating process rather than as a statistically imposed condition. To address this gap, we propose an environmentally grounded FL setup, in which client partitions are constructed according to distinct agronomic conditions rather than synthetic sampling, producing partially overlapping and locally incomplete disease distributions that mirror how disease prevalence actually varies across climate regimes. A large dataset of tomato leaf diseases is partitioned among six clients, each representing a distinct environmental region. Each participant holds only the disease classes associated with its corresponding climate regime, while some diseases naturally occur across multiple clients, reflecting the partial class overlap observed in real agro-ecological settings rather than an arbitrarily imposed statistical split. The study provides a comparative evaluation of FedAvg, FedProx, and FedAvgM under this environmentally driven heterogeneity, complemented by the integration of a stabilizer node designed to preserve global knowledge across clients with partially overlapping and incomplete label distributions. Our proposed framework for the environmentally grounded construction relies on aggregating only the shared feature-extraction backbone of the model while keeping the classifier heads fully local and decoupled from global aggregation. Several studies split the network into a shared component, aggregated by the server, and a client-specific component that remains on the device. Collins et al. [17], which learns a shared low-dimensional representation along with client-specific heads. At each communication round, clients first update their local heads while keeping the shared representation fixed and then updates the representation, which alone is averaged by the server. The opposite approach is adopted by Yi et al. [18], where clients keep their feature extractors local, and only a shared global classification header is maintained by the server. Several methods focus specifically on the classifier under non-IID data. For instance, Oh et al. [19] proposed a framework which federates only the body while keeping the randomly initialized head fixed during training and fine-tunes the head locally afterwards. FedRoD, proposed by Chen et al. [20] maintains a generic and a personalized head on top of a shared body, and CCVR, proposed by Luo et al. [21], adjusts the classifier after training using virtual representations sampled from aggregated feature statistics.

Although backbone/head decoupling has been explored in general personalized federated learning, its combination with an environmentally grounded, partially overlapping label distribution and a dedicated stabilizer node for agricultural disease diagnosis constitutes, to the best of our knowledge, a setting not previously addressed in the plant disease FL literature, which typically rely on synthetic IID or Dirichlet-based partitions.

This design allows each farm to maintain a specialized classifier adapted to its local disease profile and label distribution while still benefiting from globally shared, climate-agnostic visual representations. To verify the performance of the chosen architecture, a proprietary dataset was used, which was distributed across two clients during the training process.

Our work is organized as follows: Section 1 presents the introduction and related work, outlining the conceptual foundations and prior advances in plant disease federated systems. Section 2 details the materials and methods, including the configuration of the client dataset, the federated framework, and the experimental protocol. Section 3 reports the empirical results across all clients and algorithms. Finally, Section 4 concludes the study and discusses avenues for future research.

## 2. Materials and Methods

### 2.1. Dataset

To mimic real-world production variability, the dataset was distributed among six clients, each representing a unique agronomic context. This setup is intended to reflect the class-imbalanced and non-IID conditions, typically observed across decentralized agricultural systems. The assignment of disease classes to the participants is grounded in documented epidemiological patterns and known co-occurrence within specific production environments. Organizing the dataset into distinct client partitions enables the experimental framework to accurately reflect real-world constraints, in which data remain siloed and exhibit substantial variation in class composition.

Our work uses both public datasets and a proprietary dataset containing real-field images. The public dataset is owned by [22] and consists of images captured under perfect laboratory conditions. It contains 10 disease classes, and a few thousands of images per class, which supports the assignment of a consistent data sample to each participant during the training process. Although the public dataset is a strong start for our experiment, it is not sufficient to claim that the proposed infrastructure would achieve the same performance in a real-world scenario; therefore, we included an additional proprietary dataset to validate our results.

**Figure 1:**
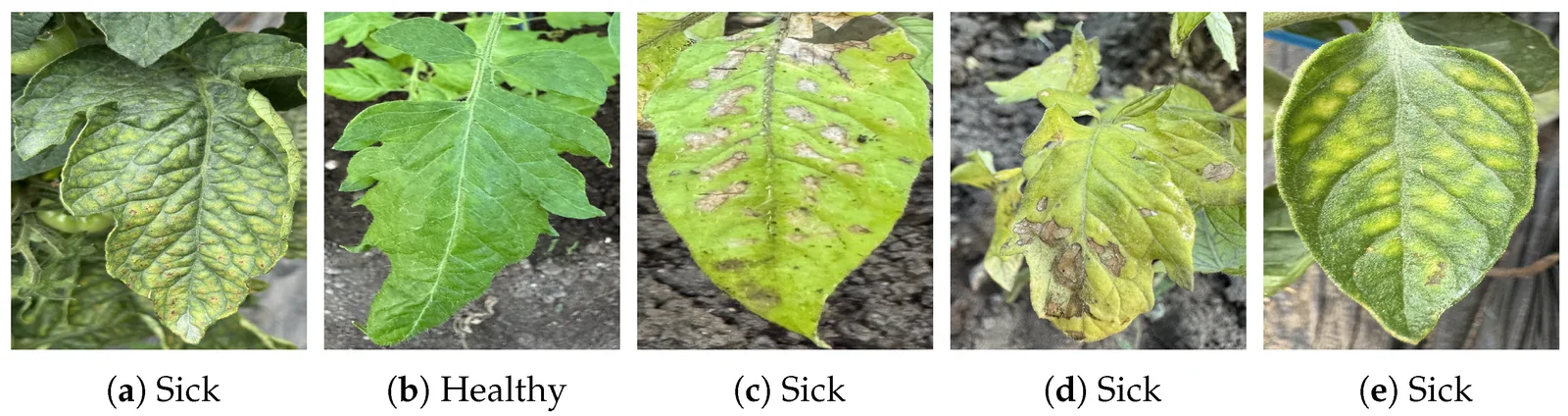
Field images.

The private dataset contains 5 disease classes, all of which are also represented among the 10 disease classes included in [22]. The images were taken in different geographical areas, during different seasons and stages of growth. Unlike the public dataset, our proprietary dataset contains imperfect images heavily affected by natural conditions, such as the presence of pests, background foliage, or even traces of treatments applied by farmers. A sample of images from the proprietary dataset can be seen in Figure 1.

The private dataset images were collected in commercial and smallholder fields in Romania, covering two distinct geographical settings: plain areas and hilly areas. Images were acquired over two growing seasons, between 2024 and 2025, and at different phenological stages of the crop, so that the dataset reflects the variability of symptom appearance over the growth cycle and across seasons. Two acquisition devices where used: a smartphone and a digital camera. Annotation was performed manually, based on the field diagnoses provided by the farmers who manage the sampled crops. The proprietary dataset specifications is shown in Table 1.

**Figure 2:**
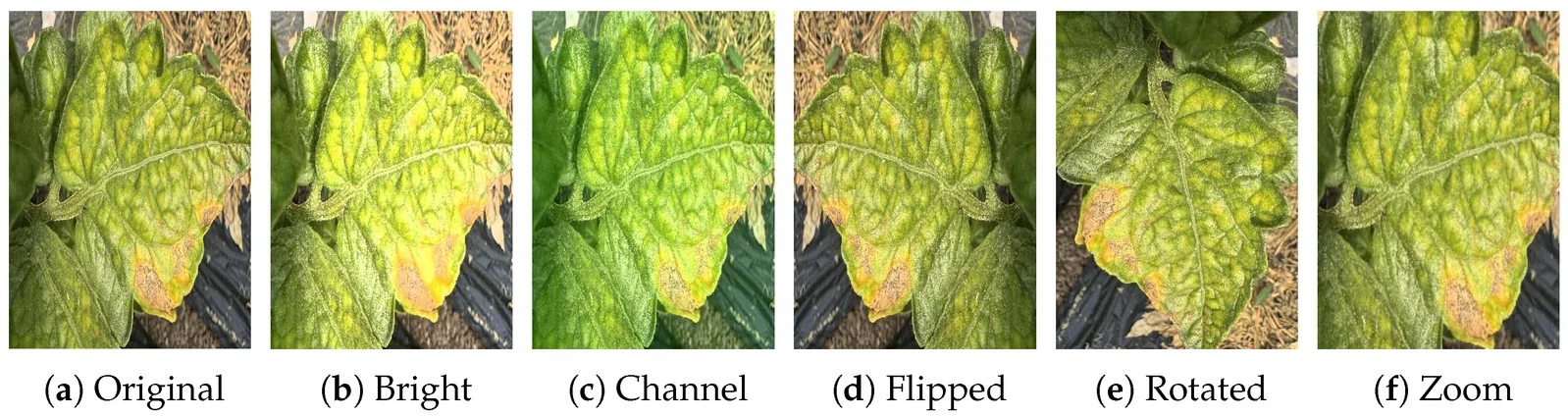
Field images after augmentation.

Since the proprietary dataset contains a limited number of images compared to the public one, several augmentation techniques were applied to increase the data samples. Based on a single image, five additional images were generated applying randomized zooming, flipping, rotation, brightness adjustment, and channel adjustment. A sample of the augmented images is shown in Figure 2.

Images from the proprietary set were used in the second part of the experiment to investigate how the established federated infrastructure behaves when the dataset of one or more clients is changed during training. The following subsections describe how the public dataset containing 10 classes was split among the 6 clients according to climate and environmental conditions.

#### 2.1.1. Client 1—Temperate, Wet Open-Field Systems

In temperate environments, where tomatoes are produced under open-field conditions, the interplay of moderate temperatures and recurrent periods of leaf-wetness creates a microclimate that strongly favors the development of foliar fungal pathogens. According to [23,24], early blight is widely recognized as one of the most problematic foliar diseases in temperate climates, being strongly influenced by rainfall frequency, recurrent dew events, and sustained canopy moisture. Similarly, septoria leaf spot is recognized as a significant foliar pathogen in temperate climates, with disease development closely associated with warm, humid conditions and frequent periods of wetness of the leaves, as documented by [25]. Given their overlapping environmental requirements and similar infection biology, the simultaneous appearance of both diseases within the same farm is highly expected. Under these considerations, *client 1* was configured to include both diseases, reflecting the characteristics of a temperate open-field tomato production. *Client 1* is an important part of our experiments because, in the second phase, it will exclusively hold field images from our proprietary dataset. The data allocation scheme for *client 1* is summarized in Table 2 and Table 3.

#### 2.1.2. Client 2—Humid, Subtropical Open-Field Systems

In humid subtropical regions, tomato crops are exposed to intense pressure from bacterial and fungal foliar pathogens. As noted by [26], the bacterial spot represents a major constraint in regions such as southeastern Brazil and Florida, due to the high humidity and sustained leaf wetness. Similarly, as documented by [27], target spot poses a serious threat, causing severe outbreaks under the warm, humid nocturnal conditions of subtropical regions. The reports of [28,29] from the University of Florida Institute of Food and Agricultural Science (UF/IFAS) note that the environmental conditions that cause target spot substantially overlap with those that favor bacterial spot, contributing to their co-occurrence in fields. Because bacterial spot, target spot, and early blight share a strong dependence on warm temperatures, recurrent leaf wetness, and sustained humidity, their co-occurrence on the same farm is highly possible. Giving this alignment in climatic requirements, bacterial spot, early blight, and target spot were selected as the representative distribution for *client 2*. The data allocation scheme is summarized in Table 4.

#### 2.1.3. Client 3—Greenhouse and High-Tunnel Production Systems

Compared with open-field cultivation, protected environments, such as greenhouses and high tunnels, create distinct climatic regimes. These environments typically maintain high humidity, reduced air movement, and increased plant handling during routine cultural practices. Under these conditions, leaf mold becomes a major limiting factor, as its infection is strongly dependent on relative humidity levels exceeding approximately 85%, according to [30]. Mosaic viruses are commonly encountered in greenhouse production systems, where the dominant mode of mechanical transmission promotes rapid spread through crop handling, worker activity, and contaminated surfaces, as noted by [31]. Although not related in their biology, these fungal and viral pathogens are jointly favored by high humidity, limited ventilation, and frequent plant contact. These environmental and operational factors justify the grouping of mosaic virus and leaf mold within the same client to accurately reflect the typical disease pressures of protected cultivation. The data allocation scheme for *client 3* is shown in Table 5.

#### 2.1.4. Client 4—Stabilizer

This client was designed to function as a stabilizing node within the FL framework, representing an institutional entity such as a research station or diagnostic laboratory. Unlike individual farms, which typically exhibit narrow and highly skewed class distributions, diagnostic centers receive samples from diverse regions. In the context of FL, this balanced client plays an important role in mitigating extreme non-IID behavior introduced by the environmental constrained clients. *Client 4* was configured to contain all the ten class diseases in approximately the same equal proportion, with the allocation schema shown in Table 6.

#### 2.1.5. Client 5—Warm Regions with High Vector and Pest Pressure

Tropical and subtropical tomato production systems are frequently exposed to simultaneous pressures from arthropod pests and insect vectors. One of the most viral pathogens transmitted in these climates remains yellow-leaf-curl. According to [32], this disease has an epidemic development closely related to the rapid growth of the vector population at elevated temperatures. Similarly, two-spotted-spider-mite exhibits thermal sensitivity, achieving rapid population growth under hot, dry conditions, as shown by [33]. Because these climatic conditions favor the co-occurrence of mosaic virus, two-spotted-spider-mite and yellow-leaf-curl, these classes were combined within the same client to represent the complex characteristics of warm tomato-growing regions. The data allocation scheme for *client 5* is shown in Table 7.

#### 2.1.6. Client 6—Highland and Maritime Climates

Highland and maritime production zones provide highly favorable conditions for late blight, as this pathogen spreads under cold temperatures and sustained periods of leaf wetness, or elevated relative humidity, according to [34]. These regions often remain largely disease-free until a sequence of weather events creates optimal conditions for infection, resulting in sudden and severe outbreaks. As a result, records from these regions typically show a predominance of healthy leaves interrupted by sudden occurrences of late blight, as shown by [35]. To mirror this empirical distribution, we associated late blight with a comparatively large portion of healthy samples in *client 6*. The data allocation scheme for *client 6* is shown in Table 8.

Along with *client 1*, *client 6* also has an important role in our experiments because, in the second scenario, it will contain exclusively field data. The distribution of the proprietary dataset for *client 6* is shown in Table 9.

### 2.2. Methodology

To evaluate FL behavior under the environmentally driven, non-IID agricultural data distribution introduced above, we analyze three aggregation strategies that represent conceptually distinct approaches to decentralized model training: Federated Averaging (FedAvg) [12], Federated Proximal (FedProx) [36], and Federated Averaging with Server Momentum (FedAvgM) [37]. These methods were deliberately selected because each addresses the client-drift problem—the tendency of locally trained models to diverge under heterogeneous label distributions—through a fundamentally different mechanism. FedAvg performs unweighted parameter averaging without any explicit correction for heterogeneity, and therefore serves as the baseline against which our environmentally grounded partitioning is stress-tested. Due to its simplicity, though, this seems to be the preferred strategy in the literature. FedProx introduces a proximal regularization term that constrains local updates to remain close to the global model, directly targeting the divergence induced by clients holding partially overlapping and incomplete disease label sets. FedAvgM incorporates server-side momentum into the aggregation step to stabilize convergence across communication rounds when client updates are inconsistent across climate regimes. Each of these strategies therefore balances the trade-off between local client adaptation and global model coherence in a different way—a trade-off that is particularly critical in our setting, where client heterogeneity reflects real agro-ecological conditions rather than an artificially imposed statistical split. The following subsections describe the theoretical motivation and original formulation of each method, together with its specific relevance to the environmentally grounded, partially overlapping federated scenario evaluated in this study.

#### 2.2.1. Federated Averaging (FedAvg)

FedAvg was introduced by [12] and represents the foundational optimization algorithm for cross-device FL. This method integrates multiple iterations of local stochastic gradient descent (SGD) performed independently on each participant with periodic global aggregation executed by a central server. During each communication round *t*, a specific subset of clients $K_{t}$ receives the global model $w_{t}$ and performs several epochs of local training on their private dataset. After completion of the previous step, each participant sends its locally updated model $w_{t+1}^{k}$ back to the server. The server then updates the global model by computing a weighted average of the received client parameters (weights), where the weights are proportional to the relative number of samples on each client. Ref. [12] proposed the following formula:

(1) $$w_{t+1}=\sum_{k\in K_{t}}\frac{n_{k}}{N}w_{t+1}^{k},$$

with $n_{k}$ denoting the number of local samples on the client *k* and $N=\sum_{k\in K_{t}}n_{k}$. On each client, local training follows the standard SGD update rule, according to [12]:

(2) $$w_{t+1}^{k}=w_{t}-\eta \sum_{i=1}^{E}\nabla F_{k}\left(w_{t};\;\xi_{i}\right),$$

where η denotes the learning rate, *E* is the number of local epochs, and $\xi_{i}$ corresponds to mini-batch subsets drawn from the private data distribution of the client.

FedAvg remains the standard reference method due to its low communication overhead, simple update structure, and stable convergence properties. Despite its simplicity, FedAvg becomes sensitive to severe data heterogeneity, local updates diverge significantly from the local objective. Although these limitations exist, FedAvg was included in our experiments because it remains the baseline in FL, providing an essential reference point against which more advanced methods can be objectively evaluated.

#### 2.2.2. Federated Proximal (FedProx)

To address the limitations of FedAvg under highly non-IID conditions, Ref. [36] introduced FedProx, which augments the local optimization objective with a proximal regularization term. In FL systems, where clients have different data distributions or highly unbalanced class subsets, local model updates can drift away from the direction of the global objective, leading to instability or biased convergence. FedProx mitigates the phenomenon of client drift by constraining local optimization steps to remain closer to the global model $w_{t}$, which in turn enhances training stability and improves robustness in highly non-IID federated environments.

Following the FedProx formulation introduced by [36], the standard local optimization problem is augmented with a proximal term that penalizes deviations from the current global mode $w_{t}$, with the modified objective defined as:

(3) $$\min_{w}\;F_{k}\left(w\right)+\frac{\mu }{2}{∥w-w_{t}∥}^{2},$$

where $F_{k}\left(w\right)$ represents the empirical loss on client *k* and μ≥0 controls the strength of the proximal constraint. This additional term restricts the extent to which local updates can drift away from the global model, thereby stabilizing the optimization under heterogeneous client data.

As also described by Li et al. [36], each client *k* minimizes in round *t* the local objective $F_{k}\left(w\right)+\frac{\mu }{2}{∥w-w^{t}∥}_{2}^{2}$, where $w^{t}$ is the global model received at the start of the round. Starting from $w_{k}^{t,0}=w^{t}$, the resulting update rule is

(4) $$w_{k}^{t,s+1}=w_{k}^{t,s}-\eta \left(\nabla F_{k}\;\left(w_{k}^{t,s};\;\xi_{k}^{t,s}\right)+\mu \left(w_{k}^{t,s}-w^{t}\right)\right),\;s=0,\ldots ,S-1,$$

where $w_{k}^{t,s}$ is the local model of client *k* after *s* local steps, $\xi_{k}^{t,s}$ is the mini-batch sampled at step *s*, η is the learning rate and *S* is the number of local steps. Both gradient terms are evaluated at the current local iterate $w_{k}^{t,s}$, while $w^{t}$ is kept fixed throughout the round. The proximal term $\mu (w_{k}^{t,s}-w^{t})$ pulls the local model back towards the global model, which limits client drift and encourages local training to remain consistent with the global objective.

This mechanism is beneficial to our setup, where clients have highly imbalanced disease classes. By reducing client drift, FedProx yields more stable convergence and a more consistent approximation of the federated solution across heterogeneous nodes.

#### 2.2.3. Federated Averaging with Server Momentum (FedAvgM)

FedAvgM, introduced by Hsu et al. [37], extends FedAvg by incorporating a momentum term at the server level in order to stabilize optimization under heterogeneous client updates. When client datasets differ significantly in class composition, dataset size, and resulting gradient dynamics, the aggregated updates tend to vary sharply across rounds, causing noticeable fluctuations between successive training rounds. Such oscillations can slow lead to unstable training trajectory.

FedAvgM addresses this variability through the use of a server momentum vector $v_{t}$, which integrates information from previous steps. In this way, the abrupt shift in the aggregated gradients is damped, promoting a smoother and more reliable global optimization trajectory. Following the formulation of [37], the momentum update is given by

(5) $$v_{t+1}=\beta v_{t}+\left(w_{t+1}-w_{t}\right),$$

where β∈[0.1) controls the contribution of past updates. The global model is then updated using the following formula

(6) $$w_{t+1}=w_{t}+v_{t+1},$$

which smooths abrupt variations arising from inconsistent client contributions, as presented in the original paper by [37].

In the context of our heterogeneous agricultural dataset, where clients differ not only in sample size but also in disease class availability, FedAvgM provides a mechanism for stabilizing the global learning process. By reducing the noise in the aggregation updates, FedAvgM improves the training stability and provides a robust comparison point alongside FedAvg and FedProx.

### 2.3. Model Description

The model employed in this work is a lightweight convolutional neural network (CNN), explicitly designed to balance computational efficiency and representational capacity—a requirement that is especially critical in cross-device FL scenarios, where each communication round requires transmitting model parameters over potentially constrained network links. This design choice is directly aligned with the backbone/head decoupling introduced in our framework: since only the feature-extraction backbone is exchanged and aggregated across clients, minimizing its parameter count directly reduces the per-round communication overhead of the federated protocol, without compromising the capacity of the locally retained classifier heads to specialize on client-specific disease profiles. Accordingly, the architecture consists of a minimalist feature-extraction backbone, shared and aggregated across all clients, and an attached classification head that remains local to each client. The model is implemented using the TensorFlow/Keras framework [38].

The backbone begins with an input layer of size 320×320×3, followed by a sequence of convolutional blocks of progressively increasing depth. The initial convolution applies 16 filters of size 3×3 with ReLU activation and same padding to retain spatial detail in the early feature maps. A max-pooling layer follows to reduce dimensionality and enhance translation robustness. The second convolutional stage increases the channel count to 32 and incorporates dilated convolutions, thereby expanding the effective receptive field without incurring additional computational cost. A final 64-channel convolutional layer further refines the learned representations, after which global average pooling aggregates spatial information into a compact feature vector suitable for classification.

**Figure 3:**
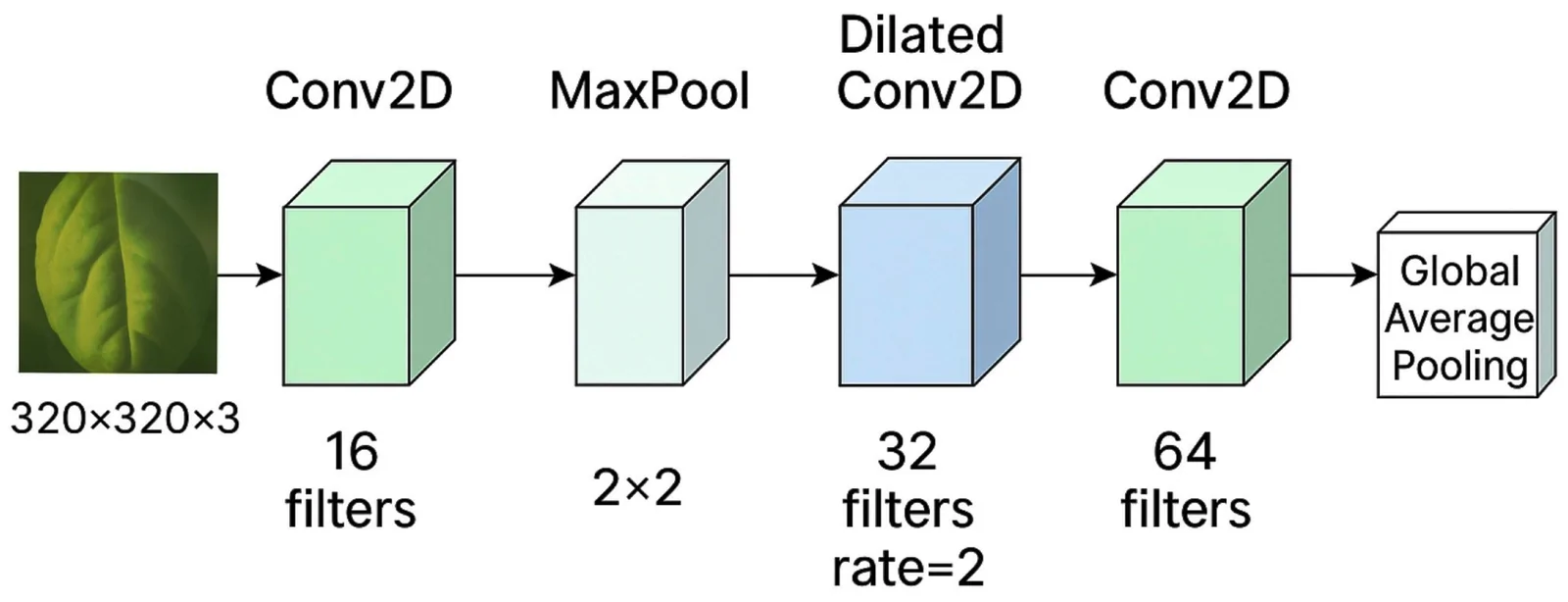
Model backbone.

The model concludes with a classification head that incorporates a dropout layer for regularization and a dense softmax output layer sized according to the number of target disease categories. This design allows the convolutional backbone to act as a shared feature extractor across all federated clients, while the classification head provides the task-specific mapping required for global inference. The structure of the model can be viewed in Figure 3.

### 2.4. Architecture Description

Our architecture belongs to the shared-body, local-head family, since only the convolutional backbone is uploaded and aggregated, while each client trains its classification head locally, first with the backbone frozen and then jointly with its last convolutional layer. The novelty of the architecture stands in the fact that we study it under a heterogeneity structure that differs from the benchmarks commonly used to evaluate such methods. Participants in the training process differ simultaneously in their class sets, in their data, and in the size of their classification task, with partially overlapping disease classes, as dictated by the agro-climatic conditions of each region. Head-sharing schemes are not applicable in this structure, as classifiers with different output dimensions and differently assigned output units cannot be averaged element-wise. Non-IID FL is usually studied under label-proportion skew generated by Dirichlet sampling, in which most clients observe most classes. Settings in which clients hold incomplete or mismatched label sets have received less attention, and existing approaches address them by modifying a shared classifier, for example through restricted softmax [39] or logit calibration [40]. Another strategy is to ensure that the federation is exposed to all classes. Zhao et al. [41] distribute a small globally shared dataset containing examples of every class to all clients, while entropy-based client selection proposed by Lutz et al. [42] chooses, in each round, the subset of clients that maximizes the class coverage of the aggregate. Our stabilizer node pursues the same goal by different means, since it is an ordinary participant that holds the complete disease taxonomy and takes part in every round. The data held by the stabilizer never leaves the node, it is aggregated with the same rule as other clients, and its influence reaches the remaining clients only through the shared backbone. The stabilizer corresponds naturally to an institution or agricultural research organization, that maintains a curated multi-region reference collection.

### 2.5. Experimental Setup

To simulate a scenario as close to reality as possible and to conduct a realistic performance evaluation of the proposed algorithms—using both ideal laboratory data and real-world data—we proposed an infrastructure consisting of six clients and a server. Five of the six clients are configured with diseases specific to certain geographic regions, as described in Section 2.1 of the current document, and one acts as a stabilizer during the training process, ensuring balance among participants. We proposed that each client maintains the classification head and only sends the backbone back to the server for aggregation. Since each client has a different number of classes and also different diseases, we investigated two possible training and aggregation scenarios.

In the first scenario, we started from the assumption that all clients are configured in the same way, regardless of the number of diseases they own. Each client runs exactly 4 epochs locally before aggregating the head, and all participants have the exact value for the head and the finetune parameters. This step is repeated for five rounds and the aggregation is triggered after all participants finish one training round.

The second scenario was built based on the results obtained in the first scenario. We realized that using the same head value for each client, as well as training for the same number of local epochs, could lead to poorer results for certain clients, since the head has to learn a more complex decision boundary. Starting from this observations, we allowed the number of head-training and fine-tuning to vary according to the client settings. Specifically, the values selected for E_HEAD and E_FINETUNE were chosen using a principled heuristic that scales approximately with the number of classes, according to Table 10.

In both scenarios, the three aggregation algorithms—FedAvg, FedProx, and FedAvgM—were executed. Given the significant improvement in results observed when transitioning from the configuration used in the first scenario, an additional set of three training rounds was introduced in the second scenario. In the last three rounds, the dataset of two clients was completely replaced with real field data from the proprietary dataset, by maintaining the exact classes present in the first five rounds, and the remaining four clients were left unchanged. In this way, we analyzed the impact of real field data ingestion in a federated setup trained exclusively on perfect laboratory images.

## 3. Results

We now present the experimental results obtained across all participating clients for the three federated optimization algorithms implemented in this study: FedAvg, FedProx, and FedAvgM. In the first scenario, each algorithm was executed for five global communication rounds, during which clients performed a fixed number of 4 training epochs per round. In the second scenario, each algorithm was executed for a variable number of local training epochs (between 6 and 12) per round. This range provides a controlled environment for comparing algorithmic behavior while respecting the computational constraints of the simulated distributed infrastructure. Crucially, because client heterogeneity in our setup arises from real agronomic and climatic conditions rather than an artificially imposed statistical partition (e.g., Dirichlet-based label skew), the algorithmic behavior observed below reflects how these methods perform under a genuinely difficult, ecologically grounded non-IID scenario—a setting that, to our knowledge, has not been previously used to stress-test FedAvg, FedProx, and FedAvgM in the plant disease FL literature. This is precisely what makes the per-client divergence reported the following scientifically meaningful rather than an artifact of synthetic sampling.

### 3.1. Temperate Wet Systems (Client 1)

Table 11 presents the evolution of *client 1* validation accuracy over five communication rounds for each algorithm, in the first scenario. FedProx demonstrates the most consistent high performance across training rounds in this scenario, suggesting that the proximal term effectively mitigates client drift for this particular data distribution—though its accuracy also drifts gradually downward from round 3 onward (0.9225 to 0.8963), a pattern that recurs across several clients and is discussed further below. FedAvg shows moderate fluctuating accuracy without a clear trend, illustrating the difficulty of achieving stable convergence under unweighted averaging alone. Instead, FedAvgM improves progressively, closing much of the gap with FedProx by later rounds. Taken together, these results already illustrate a central difficulty of this study: no aggregation strategy is uniformly reliable, and each carries a distinct failure mode under realistic heterogeneity.

**Figure 4:**
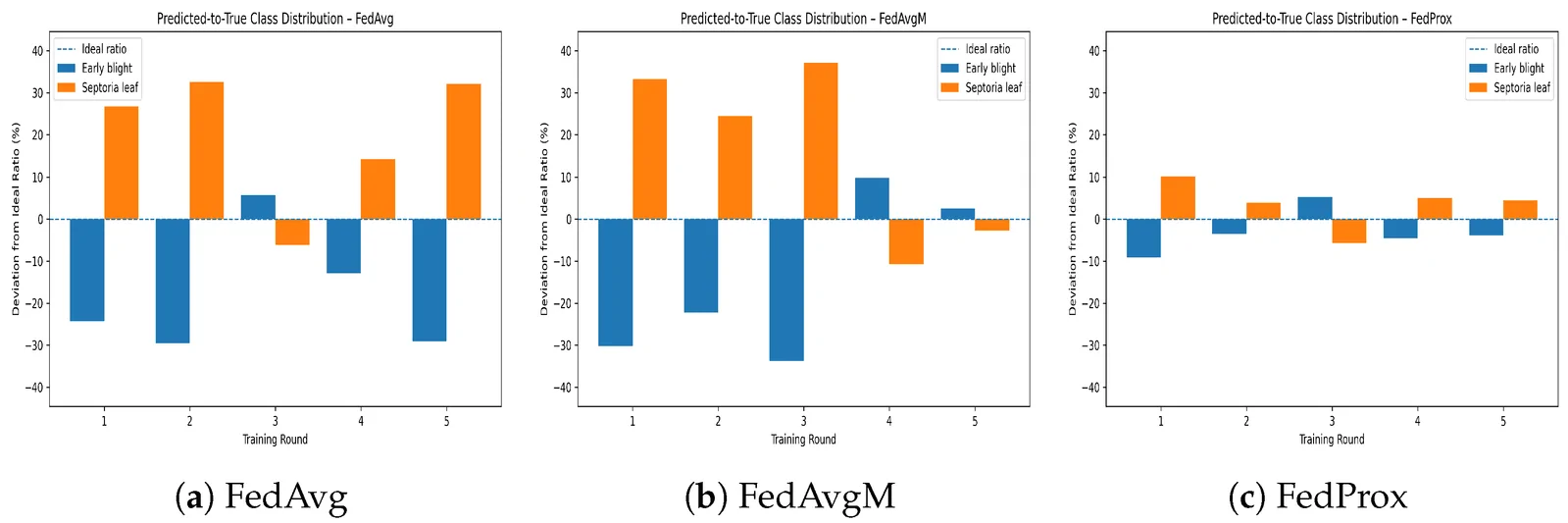
Predicted to true class distribution for *client 1* during the first scenario.

As we can observe in Figure 4, the predicted-to-true class distribution for *client 1* reveals substantial differences in the stability of the aggregation strategies. FedAvg generally under-predicts early blight and over-predicts septoria leaf across several communication rounds, while FedAvgM displays even stronger oscillations during the initial rounds, suggesting that the accumulated server momentum may amplify transient client-specific biases before convergence. In this scenario, FedProx effectively limits the local model drift, indicating a more stable preservation of class proportions compared to FedAvg and FedAvgM.

Table 12 presents the evolution of *client 1* validation accuracy over the second scenario, when eight communication rounds were run for each algorithm, on a new model, unaffected by the rounds run in the first scenario. The first five rounds were performed on the public dataset, where the three algorithms exhibit close learning dynamics, with accuracy consistently exceeding 90%. This percentage confirms the performance of our model on public datasets, aligning with other results obtained by the scientific community. In the last three rounds, the dataset for *client 1* was completely changed with the proprietary one, so that we can analyze how the system behaves on real data ingested in the middle of the training. It is important to note that we kept the same exact classes, specifically early blight and septoria leaf. As we can observe, the accuracy decreases by approximately 10%, but it still remains above 82%, which is a considerable value, given the simulated scenario. It can be noted that the first five training rounds had a significant impact on *client 1* performance, since the system continued to deliver promising results even when the data source was replaced with a considerably smaller and different set of images.

**Figure 5:**
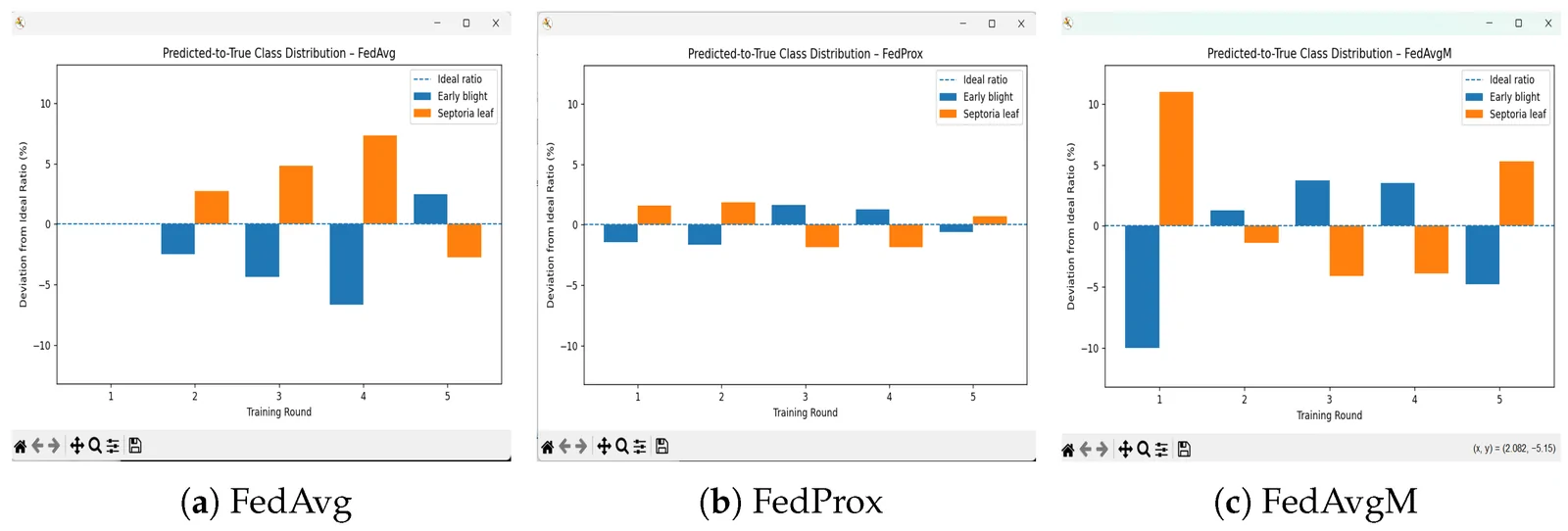
Predicted to true class distribution for *client 1* during the second scenario.

As we can observe in Figure 5, the predicted-to-true class distribution for *client 1* is significantly better compared to the first scenario for FedAvg and FedAvgM, and almost similar for FedProx. These results indicate that tailoring the number of epochs and rounds based on the number of classes held by each client can substantially improve results, even for clients possessing a small number of classes.

### 3.2. Humid Subtropical Systems (Client 2)

In the first scenario, *client 2* exhibited a markedly different convergence profile across the three optimization strategies, as seen in Table 13. FedAvg produced moderately stable performance across rounds, with validation accuracy oscillating between 0.75–0.80 and showing no strong upward trend. FedProx, despite its proximal regularization designed to reduce client drift, exhibits marginally lower performance than FedAvg and FedAvgM, with a final value of 0.7107 at the end of the final training round, reflecting the increased difficulty of its three-class local distribution—itself a direct consequence of the environmentally grounded partial label overlap introduced in our experimental design, rather than an arbitrary class count. In contrast, FedAvgM follows a substantially more favorable trajectory, rising from 0.7577 to a peak of 0.9046 before stabilizing at 0.8950. These results show that *client 2* benefits disproportionately from momentum-based aggregation, achieving both higher accuracy and smoother convergence than under FedAvg or FedProx—a finding with direct practical value, since it suggests that clients with narrow, specialized disease profiles may require momentum-aware aggregation rather than the drift-correction mechanisms typically favored in the FL literature.

**Figure 6:**
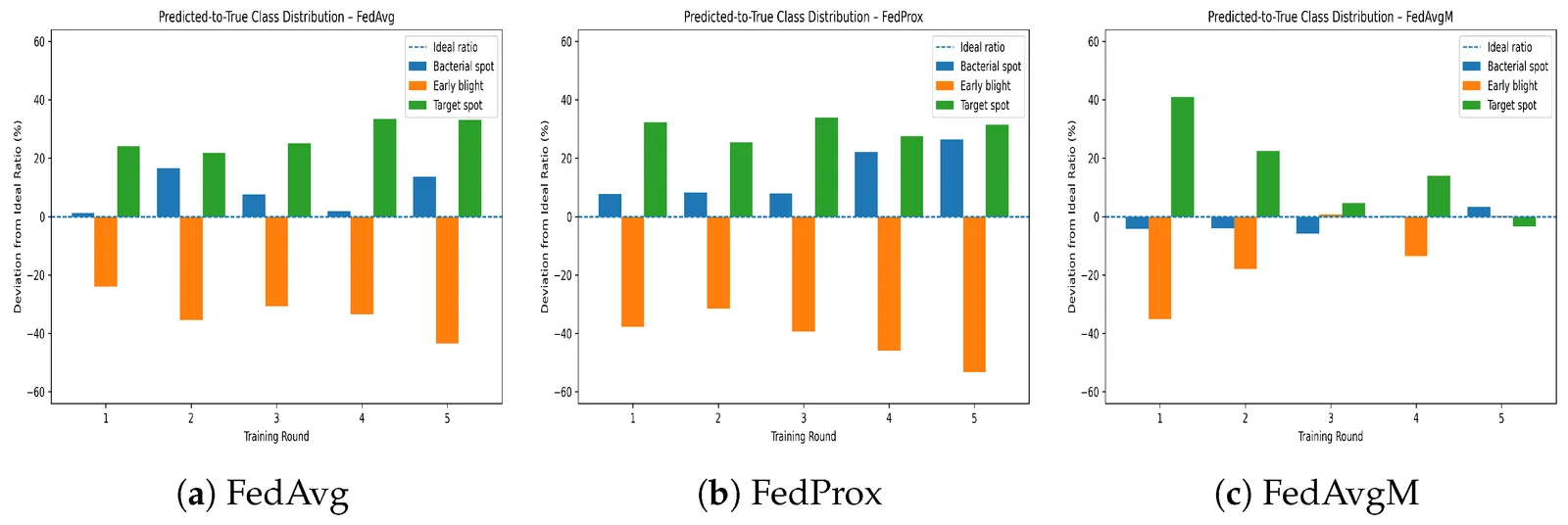
Predicted to true class distribution *client 2* during the first scenario.

As we can observe in Figure 6, in the first scenario, *client 2* struggles for FedAvg and FedProx, but has stable final results for FedAvgM. In both FedAvg and FedProx, the model under-predicts early blight, but over-predicts target spot and bacterial spot. This may be due to the fact that the two diseases—target spot and bacterial spot—can exhibit similar foliar symptoms, making visual differentiation difficult during early stages of development. Furthermore, this client has three classes, highlighting that local head training was insufficient to clearly distinguish between diseases. Unlike *client 1*, where the best results in the same scenario were obtained with FedProx, in this case, FedAvgM yielded the most stable results. This suggests that the momentum term facilitates more effective optimization for the client-specific distribution, potentially by smoothing successive global updates and reinforcing consistent descent directions across communication rounds.

In the second scenario, *client 2* exhibits a close convergence profile across the three optimization strategies, but with lower accuracy values than *client 1*, as shown in Table 14. FedAvg and FedAvgM demonstrated strong accuracy performance, starting with accuracy levels below the 80% threshold and reaching 93% and 92% after the eight training rounds. Although it achieved lower accuracy than FedAvg and FedAvgM, FedProx demonstrated steady growth throughout the training rounds and recorded a significant improvement compared to the results obtained in the first scenario. It is important to mention that, the strong upward trend for all strategies in the last three rounds is achieved because the dataset remained unchanged for *client 2*, which means that adding clients injected with real data did not negatively affect the performance of the other nodes. Another interesting observation is the fact that, although *client 1* and *client 2* share a common disease—early blight—the accuracy of *client 2* continues to improve in the last three training rounds for all strategies.

**Figure 7:**
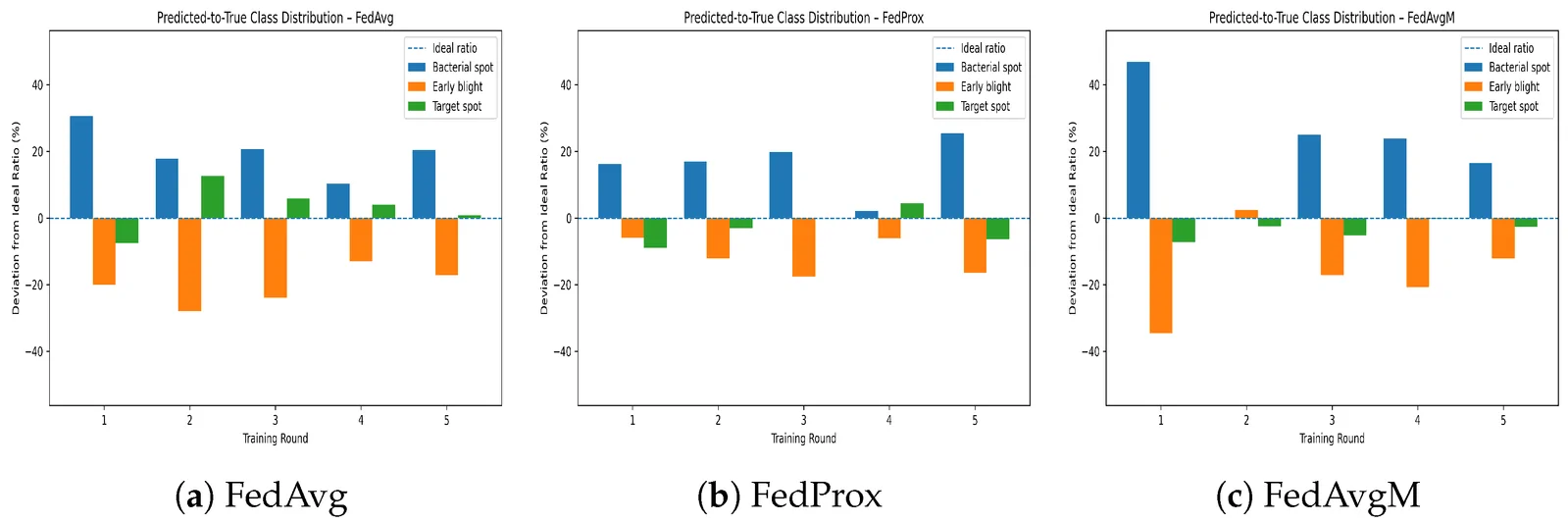
Predicted to true class distribution *client 2* during the second scenario.

As we can observe in Figure 7, the predictions for target spot and early blight improved considerably compared to the first scenario, but bacterial spot recorded a higher level of over-prediction, meaning this disease was more difficult to distinguish during the first rounds.

### 3.3. Greenhouse Production (Client 3)

During the first scenario, *client 3* shows the clearest separation in performance among the three federated optimization strategies, making it the most illustrative case for comparing algorithmic robustness. FedAvg exhibits moderate but unstable behavior, with validation accuracy fluctuating between 0.69 and 0.89 without a discernible trend. FedProx improves substantially on this instability in the early rounds, reaching 0.9041 and 0.9074 in rounds 1 and 2, though its advantage narrows over time as accuracy declines to 0.7789 by round 5—again consistent with the gradual-decline pattern observed for FedProx elsewhere in this study. FedAvgM, by contrast, achieves exceptionally strong and largely sustained results, surpassing 0.96 in four out of five rounds and peaking at 0.9880 in round 3. This client demonstrates the clearest merit of momentum-based aggregation under environmentally grounded heterogeneity: when local and global optimization signals are sufficiently aligned, FedAvgM can substantially outperform both alternatives. The evolution of *client 3* in the first scenario can be seen in Table 15.

**Figure 8:**
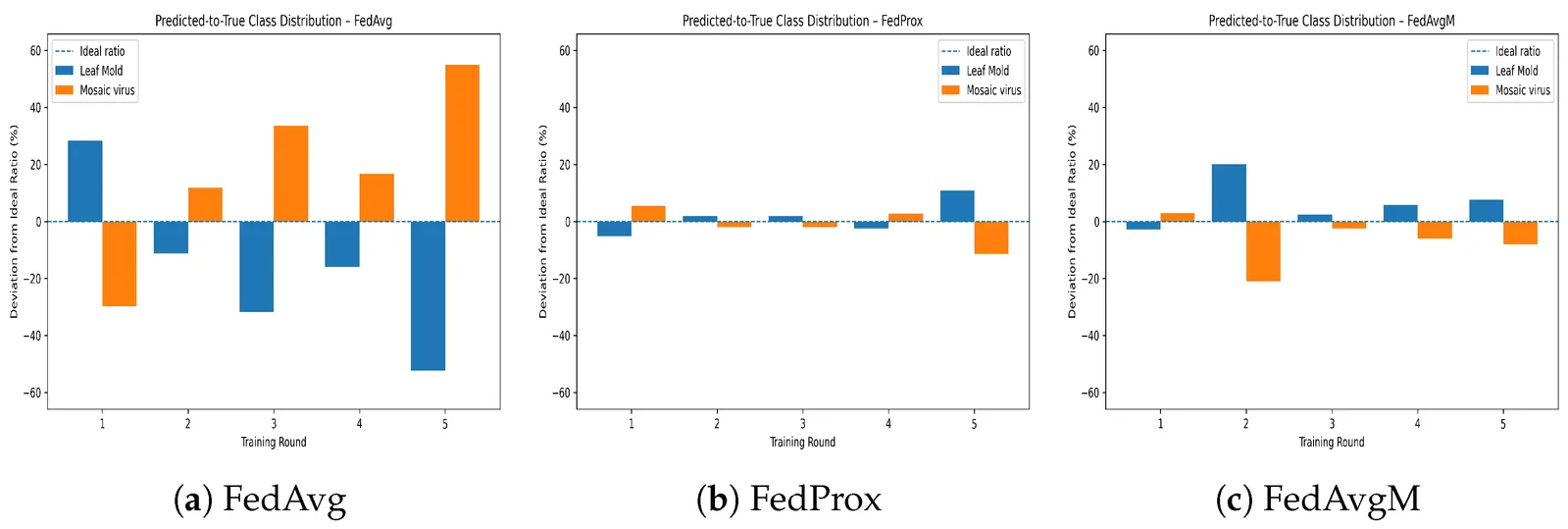
Predicted to true class distribution *client 3* during the first scenario.

As we can observe in Figure 8, FedAvgM and FedProx yield very good predictions for both classes, whereas FedAvg over-predicts mosaic virus, and under-predicts leaf mold. For *client 3*, FedAvg proved to be the most unstable strategy, a fact that supports our view that a single universal algorithm cannot work across all operational scenarios.

In the second scenario, the accuracy improved substantially for each strategy, as we can observe in Table 16. While the increase for FedAvgM is very small, FedProx and FedAvg show significant improvements, with accuracy reaching 99%. The pronounced improvement obtained in this scenario may be related to the visual characteristics of the local disease classes. Although mosaic virus and leaf mold are etiologically different, both could have partially overlapping symptoms, and an insufficient adapted representation may fail to capture the finer discriminative features which separate them.

**Figure 9:**
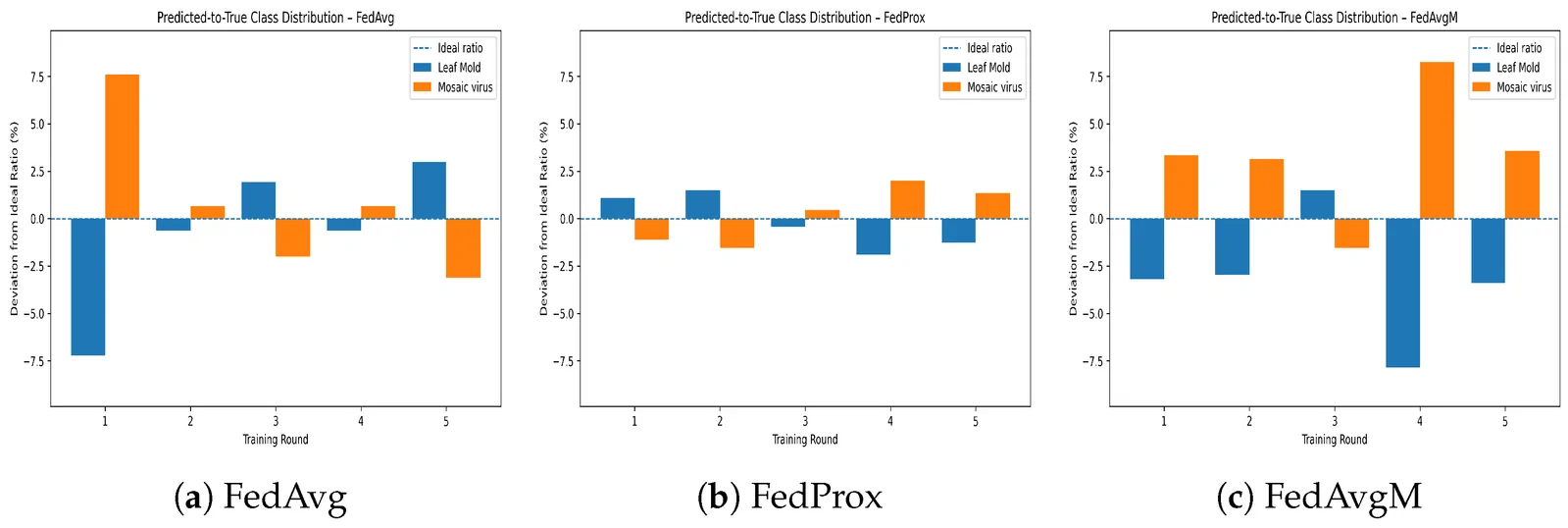
Predicted to true class distribution *client 3* during the second scenario.

As we can observe in Figure 9, compared to the first scenario, the predicted-to-true-class ratio indicates a stabilization of predictions across all strategies, although the values recorded for FedProx and FedAvgM are slightly more pronounced.

### 3.4. Full-Taxonomy Stabilizer Node (Client 4)

*Client 4* has an essential role in the proposed training scheme, as it serves as a globally representative participant, containing samples from all disease classes and contributing to each federated training round. For the first scenarios, *client 4* exhibited poor performance, as the head failed to learn sufficient features within the standard number of rounds and epochs defined for all nodes. The poor results it yielded—accuracy below 0.60 for each strategy—indicated the need to customize the training scheme, which led to the implementation of the second scenario. As shown in Table 17, the accuracy of the stabilizing node exhibited an upward trend in the second scenario, even after the integration of clients injected with real data. Selecting specific values for the E_HEAD and E_FINETUNE parameters enabled the client to learn the defining characteristics of each class, thereby increasing accuracy by approximately 20% for each strategy.

**Figure 10:**
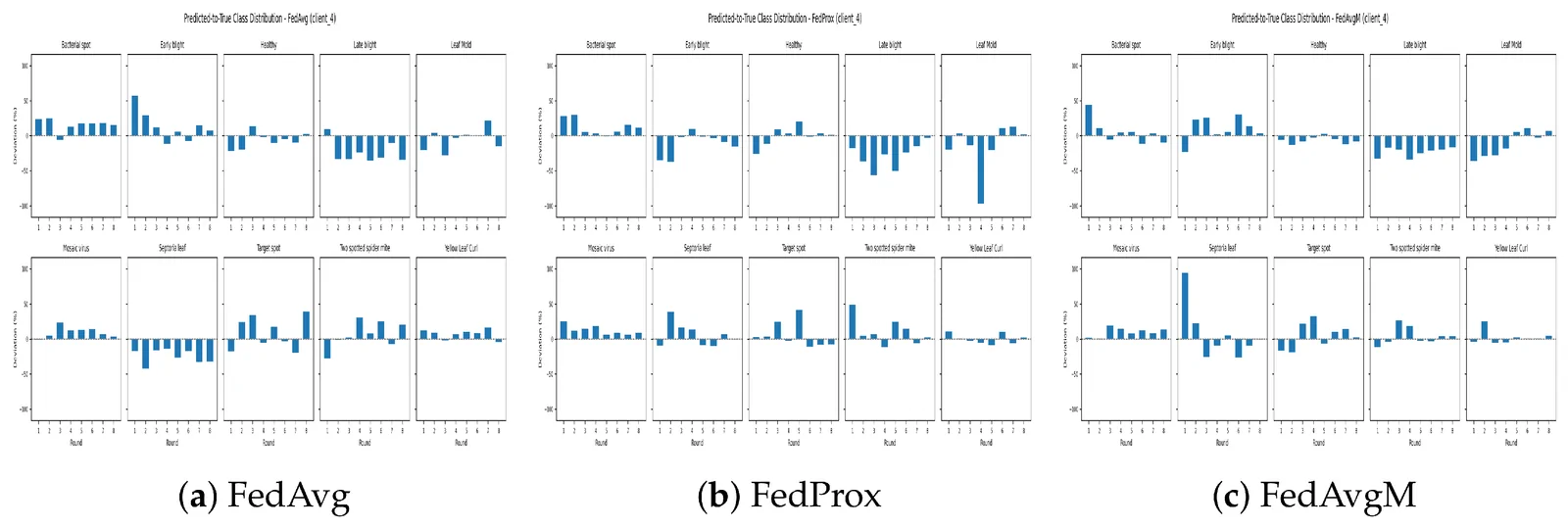
Predicted to true class distribution *client 4* during the second scenario.

Figure 10 illustrates the predicted-to-true class distribution for *client 4*, for each applied strategy. It can be observed that the stabilizing node yielded stable results throughout the training process, showing visible improvement from one round to the next.

### 3.5. Warm Regions (Client 5)

**Figure 11:**
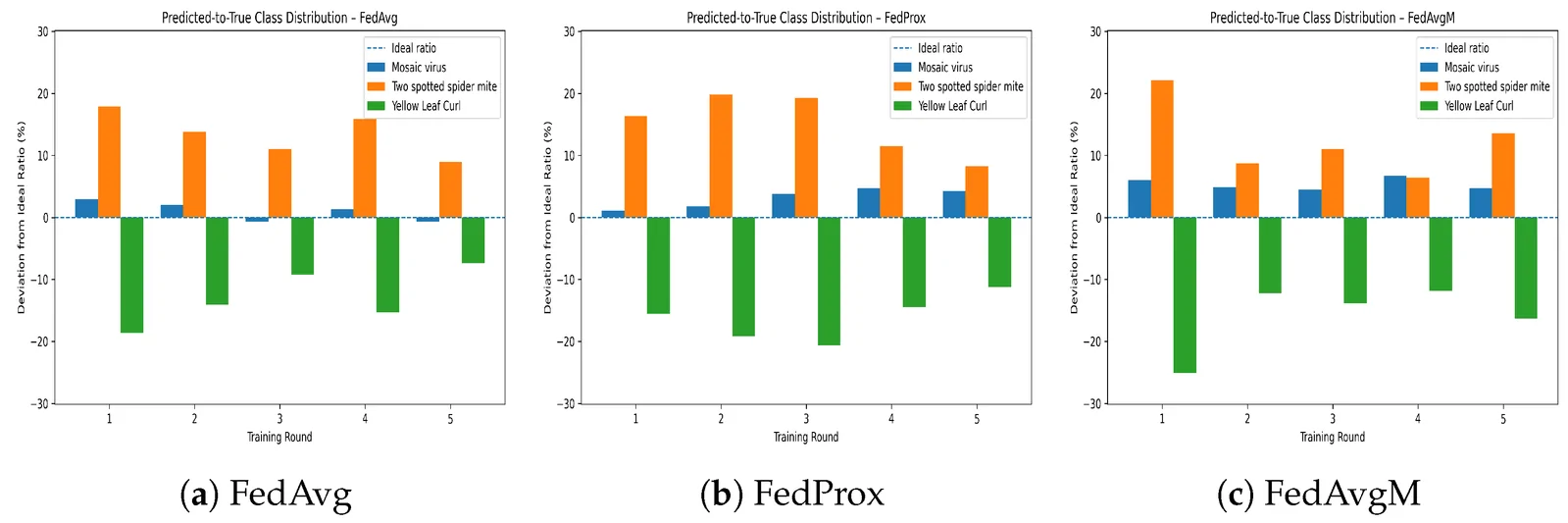
Predicted to true class distribution *client 5* during the first scenario.

In the first scenario, *client 5* exhibits consistently high validation performance across all three federated optimization strategies, standing in clear contrast to the volatility observed in *clients 1–3*, as seen in Table 18 and Figure 11. FedAvg and FedProx both show remarkably stable behavior, with FedAvg slightly leading overall and reaching its peak accuracy of 0.9286 in round 2. FedAvgM performs comparably well, with marginally higher early-round variability, starting lower (0.8813) but converging toward the accuracy level of the other two algorithms by round 2 onward. The stability observed for *client 5* is itself a meaningful result: it indicates that when a client’s local disease distribution closely aligns with the global model’s learned representation, the choice of aggregation strategy becomes largely inconsequential—a useful practical boundary condition for deploying any of the three methods in less heterogeneous agricultural regions.

**Figure 12:**
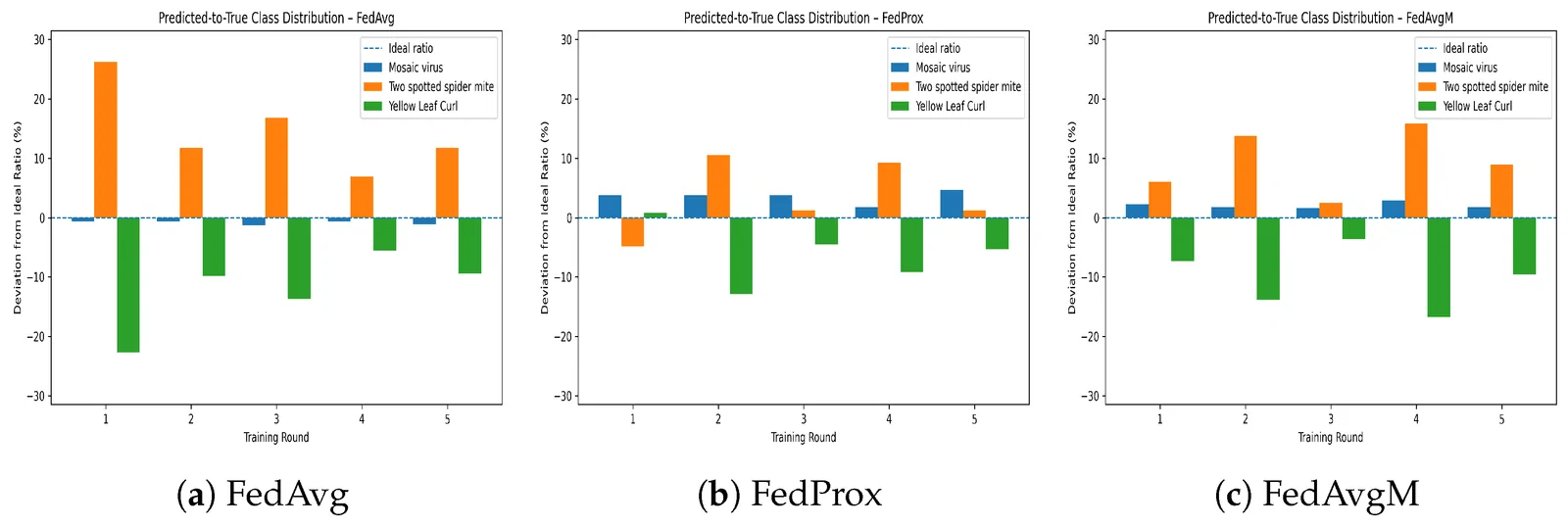
Predicted to true class distribution *client 5* during the second scenario.

Although *client 5* achieved significantly better results than the other participants in the first scenario, even better calibration was observed in the second scenario across all applied aggregation strategies. As can be seen in Table 19, an accuracy of over 93% was achieved for each algorithm, and the inclusion of clients possessing real data in the training scheme did not affect the performance of *client 5*. Also, as shown in Figure 12, *client 5* achieved stable predictions among all classes during the second scenario.

### 3.6. Highland and Maritime Systems (Client 6)

**Figure 13:**
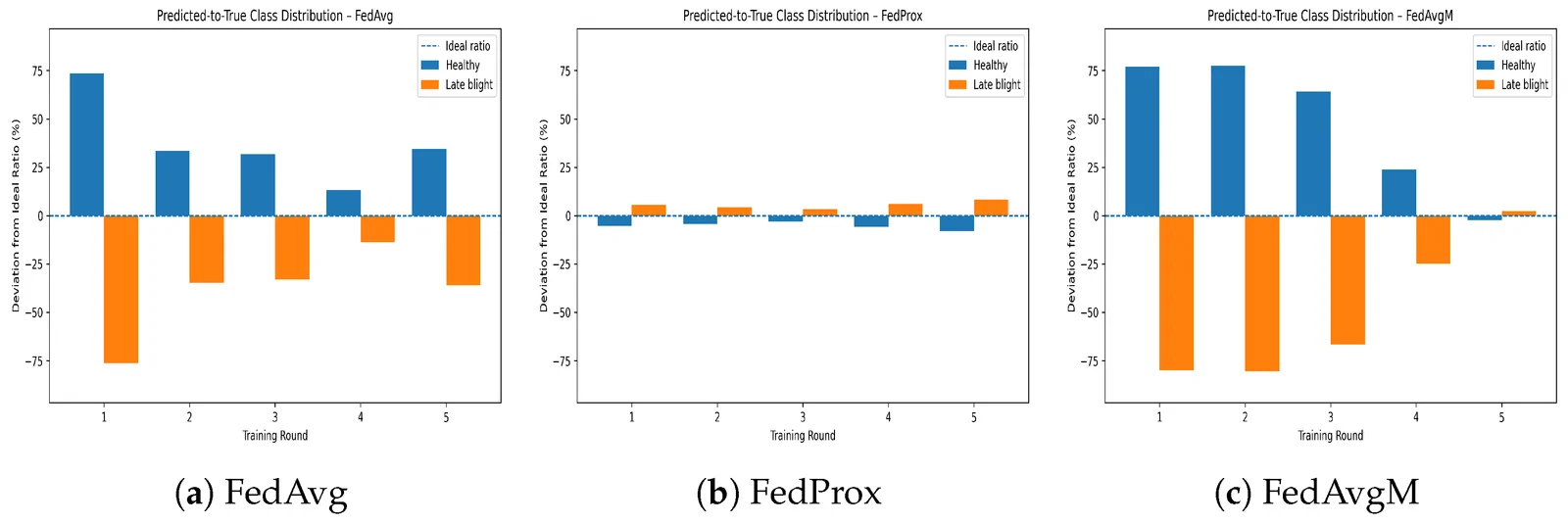
Predicted to true class distribution *client 6* during the first scenario.

In the first scenario, *client 6* presents the most challenging scenario among all participants, primarily due to the strong class imbalance that characterizes its local dataset, as shown in Table 20. This is a direct and expected consequence of the environmentally grounded partitioning strategy, in which some disease classes occur rarely or not at all within a given climate regime. This client is the sternest test of the entire experimental design, and the divergence in algorithmic behavior it exposes is arguably the most informative result of this study. FedAvg shows a gradual improvement, rising from 0.5371 in round 1 to a peak of 0.7479 in round 4, before declining slightly in the final round; this trajectory indicates that iterative aggregation progressively enhances the shared backbone’s ability to represent the client domain, although the instability across rounds reveals a clear sensitivity to label imbalance that FedAvg alone cannot resolve. FedProx, by contrast, delivers strong and comparatively stable performance, maintaining validation accuracy between 0.8824 and 0.9301. Notably, FedProx performs the best result among the three methods for this client, even though it follows the same gradual downward drift observed elsewhere. FedAvgM struggles considerably in the early rounds (0.5307, 0.5286), a clear limitation exposed by severe class imbalance, before accelerating markedly as the momentum mechanism accumulates more reliable directional information, reaching 0.7044 by round 5. This client illustrates extreme, ecologically grounded label imbalance, as shown in Figure 13, and its key merit is precisely this kind of realistic difficulty, rarely captured by synthetic non-IID partitions, that reveals meaningful and actionable differences between aggregation strategies.

**Figure 14:**
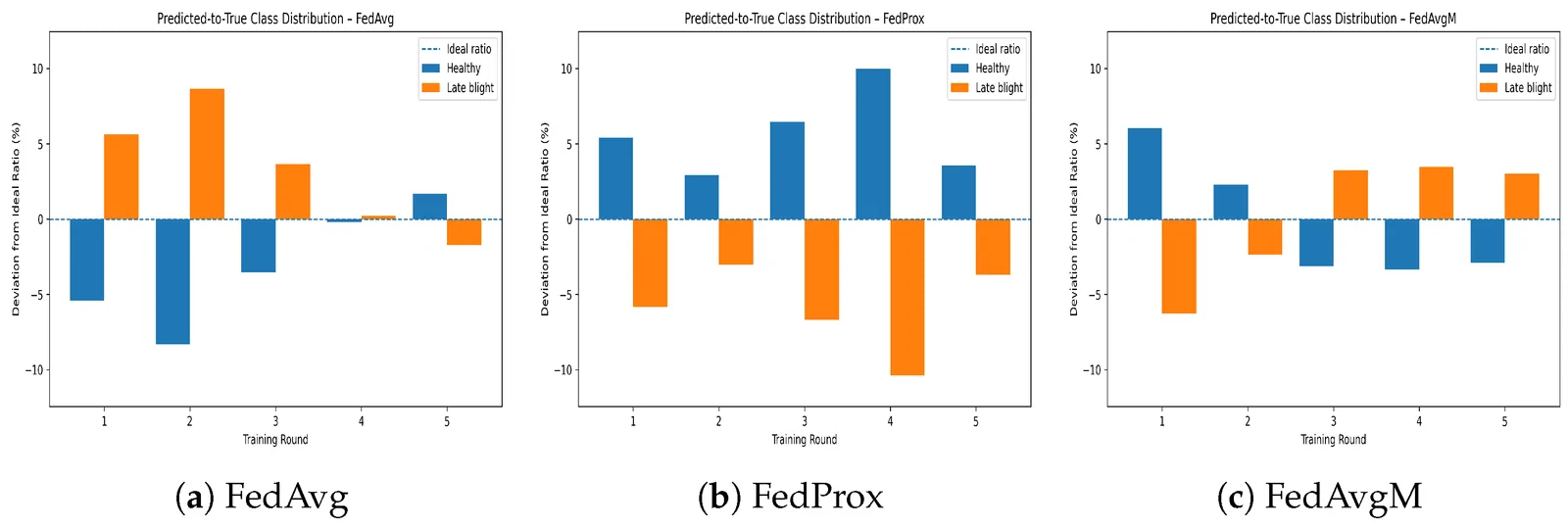
Predicted to true class distribution *client 6* during the second scenario.

*Client 6* recorded the most significant improvement when transitioning to the second scenario. As it can be seen in Table 21 and Figure 14, this client shows a strong increase in accuracy for every strategy. It is also interesting to note that *client 6* was trained exclusively on real data for the last three rounds, and the results achieved—while slightly lower than those of the first five rounds—were still superior to those of the first scenario. This highlights the importance of appropriately parameterizing each participant, despite the choice of a rigid training scheme.

### 3.7. Discussions

**Figure 15:**
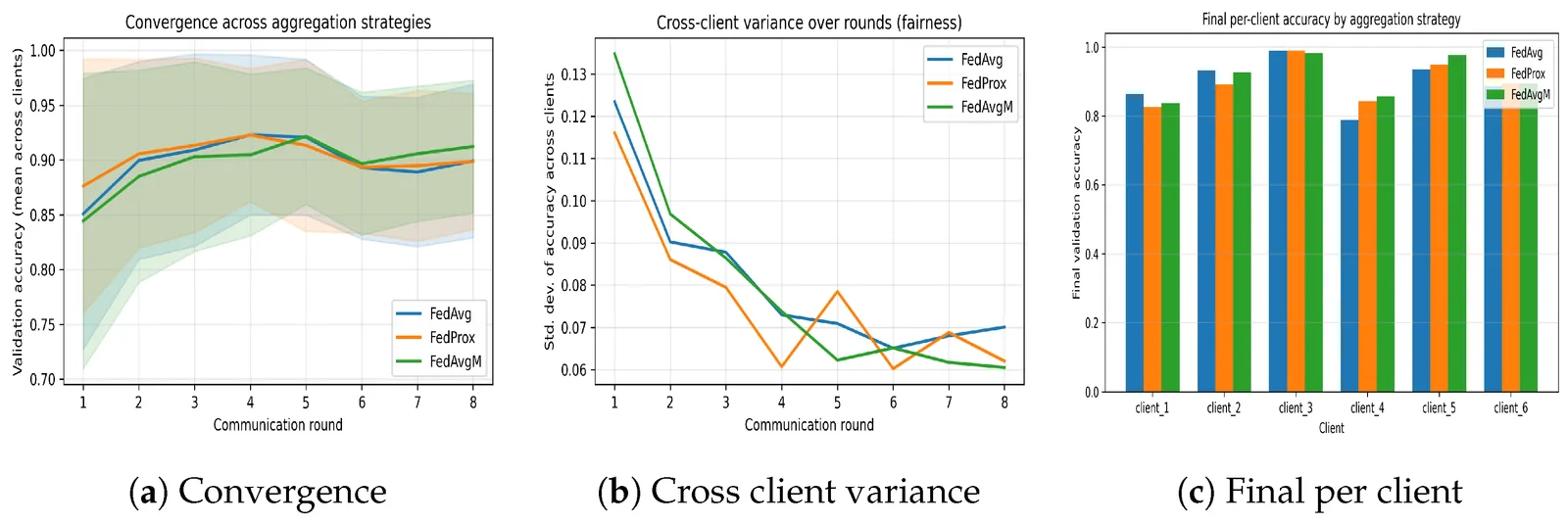
Global results.

Figure 15 presents the results obtained for all clients at the end of our experiments. A slight inflection in convergence is observable around the sixth training round—a significant point, as the influence of clients injected with real-world data begins to manifest there.

The cross-client accuracy variance decreases for all aggregation strategies, indicating an increasing alignment of predictive performance across clients. As we can observe in Figure 15b, FedAvgM initially presents the highest inter-client variability, but progressively converges toward the lowest final variance, suggesting that momentum-based aggregation may improve long-term performance. FedProx maintains relatively low variance during several intermediate rounds, whereas FedAvg shows a steadier decline before reaching a stable level.

**Figure 16:**
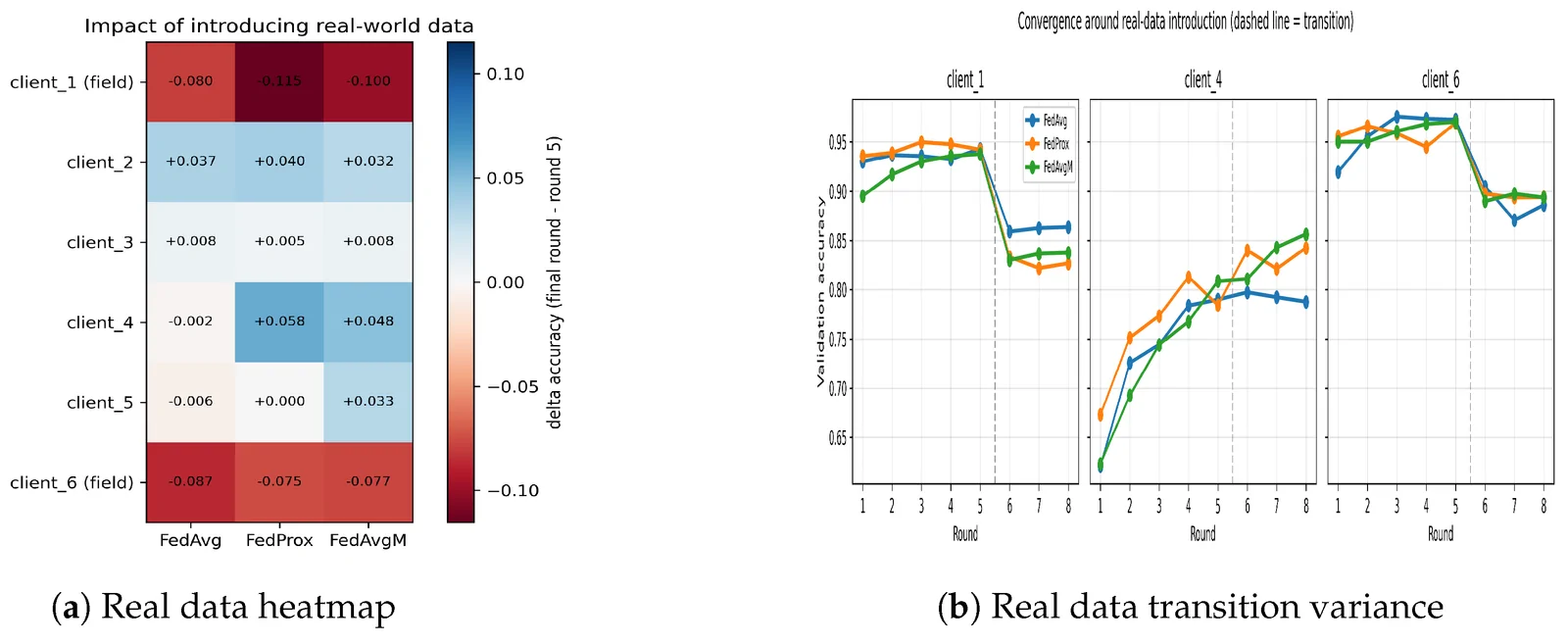
Real data impact on the performance of the system.

An interesting observation resulting from our experiments is the influence that real-world data has on a system trained on perfect data. We paid special attention to *client 1* and *client 6*—since their training dataset was replaced with data from the proprietary set for the final three training rounds—as well as to *client 4*, which acts as a stabilizer and inherently holds all classes. While the stabilizer exhibits stable predictive performance, *client 1* and *client 6* experience a decrease in accuracy across all aggregation strategies. This behavior suggests the presence of a pronounced domain shift between the newly introduced data and the previously learned representations. To quantify how the introduction of real field data affect each participant, we compute for every client k and aggregation algorithm a, the accuracy difference $\Delta_{k,a}={\mathrm{Acc}}_{k,a}\left(8\right)-{\mathrm{Acc}}_{k,a}\left(5\right)$, where ${\mathrm{Acc}}_{k,a}\left(r\right)$ is the top-1 accuracy on client k validation set after round r, and round 5 is the last round before *client 1* and *client 6* switched to field images, as seen in Figure 16. *Client 1* loses 8.0, 11.5 and 10.0 percentage points under FedAvg, FedProx and FedAvgM, respectively, and *client 6* loses 8.7, 7.5 and 7.7 points. As shown in Figure 16, this drop occurs abruptly at round 6, when the field data are introduced, and the accuracy remains approximately stable over rounds 7-8. For these two clients, Δ combines the effect of the new training data with that of a herder validation domain, since their validation images also change from laboratory to field images. In contrast, none of the clients whose own data remained unchanged shows a substantial degradation, since the largest negative change among them is −0.6 points, which is within the variability expected from the size of the validation sets. These results indicate that the domain shift introduced by the field data degrades the clients that train on it, but does not propagate as a measurable loss to the rest of the federation through the shared backbone.

To interpret the accuracy degradation of *client 1* and *client 6* from the feature perspective, we compared the backbone representations of laboratory and field images of the same five disease classes which the proprietary dataset contain. For each aggregation algorithm, we used the final global backbone of the second scenario and extracted the 64-dimensional global-average-pooled features of 150 laboratory images and 150 field images per class, sampled random with a fixed seed, so that all backbones were evaluated on the same 1500 images. We visualized the features with t-SNE and quantified the discrepancy between the two domains with three complementary measures. First, the squared maximum mean discrepancy (${\mathrm{MMD}}^{2}$) with a Gaussian kernel, using the unbiased estimator, the median heuristic for the kernel bandwidth, and a permutation test with 200 permutations. Second, the proxy A-distance, PAD=2(1−2ϵ), where ϵ is the five-fold cross-validated balanced error of a logistic-regression classifier trained to distinguish laboratory from field features. Third, for each class c, the normalized centroid shift

(7) $$s_{c}=\frac{{∥\mu_{c}^{L}-\mu_{c}^{F}∥}_{2}}{\frac{1}{2}\left({\bar{r}}_{c}^{L}+{\bar{r}}_{c}^{F}\right)},$$

where $\mu_{c}^{L}$ and $\mu_{c}^{F}$ are the class centroids in the laboratory and field domains and ${\bar{r}}_{c}^{L}$, ${\bar{r}}_{c}^{F}$ the mean distances of the samples to their centroid.

**Figure 17:**
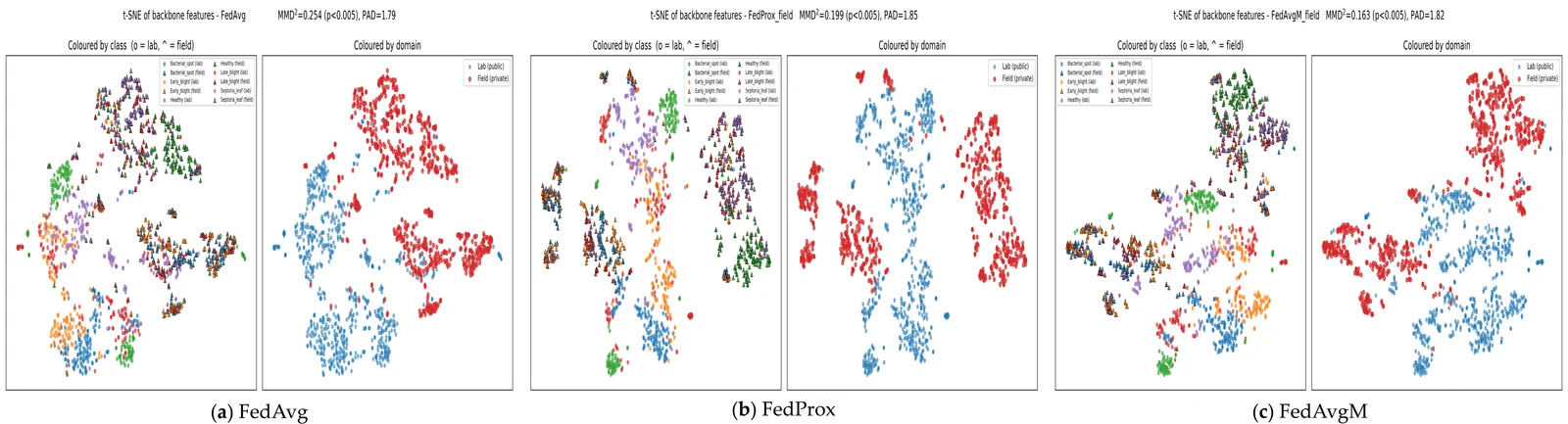
t-SNE projection of the backbone features.

As seen in Figure 17, the backbone features reveal a pronounced domain gap between laboratory and field images for all three aggregation algorithms. A linear classifier distinguishes laboratory from field features with a balanced accuracy of 0.947–0.963, corresponding to proxy A-distances of 1.79–1.85, close to the maximum value of 2. The maximum mean discrepancy is significant in all cases (*p* < 0.005), and on the t-SNE projections laboratory and field images occupy almost disjoint regions of the feature space. The magnitude of the discrepancy differs between algorithms: ${\mathrm{MMD}}^{2}$ decreases from 0.254 (FedAvg) to 0.199 (FedProx) and 0.163 (FedAvgM), and the mean centroid shift follows the same order (1.39, 1.29 and 1.07). The FedProx and FedAvgM backbones therefore bring the two domains closer on average, but not enough to make them overlap, since the domains remain almost perfectly linearly separable for all three.

The t-SNE projections show a second effect that is consistent across all three algorithms: within the field domain, the disease classes are poorly separated. Field images of Bacterial_spot, Early_blight and Late_Blight are largely intermixed, whereas healthy field leaves form a distinct group. We attribute this to the complex backgrounds of the field images (soil, neighboring leaves, mud spots, and pests), which, together with the visually similar necrotic lesions produced by these diseases, dominate the features of the lightweight backbone, in which only the last convolutional layer is updated during federated training. The accuracy degradation of *client 1* and *client 6* thus has two feature-level causes: a shift in the feature distribution of each disease away from the laboratory domain, which prevents the laboratory-trained decision boundaries from transferring, and a reduced separability of the diseases within the field domain itself.

**Figure 18:**
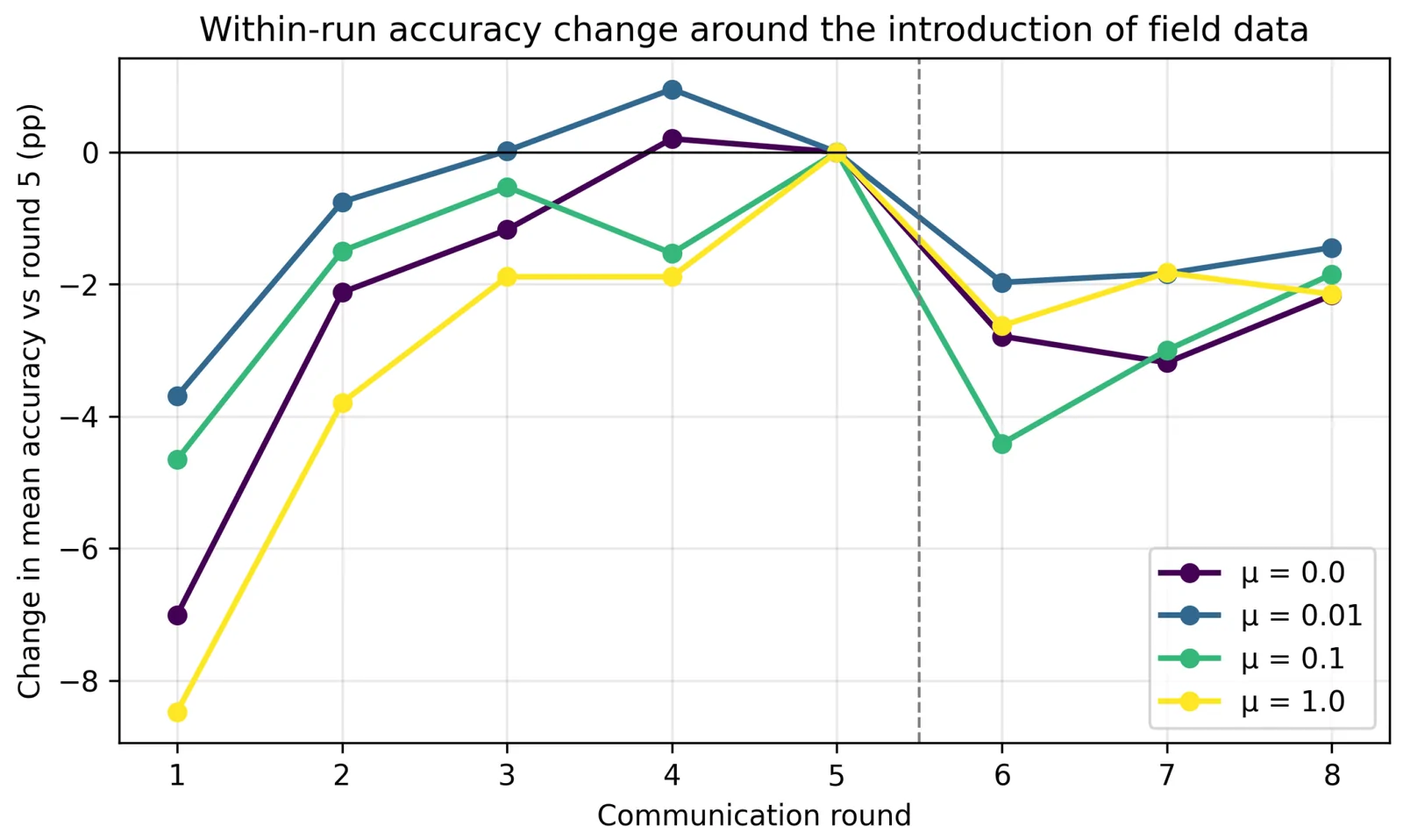
FedProx evolution.

To determine whether the late-round decline observed for FedProx is caused by the proximal term, we compared μϵ{0,0.01,0.1,1.0}, where μ=0 corresponds to FedAvg, and logged during local fine-tuning the ratio between the norms of the proximal and task gradients and the relative drift of the trainable backbone layer from the global model. The results can be seen in Table 22 and Figure 18. Because the first two backbone layers remain frozen at their random initialization, the configurations differ in absolute accuracy level, and we therefore compare them through the change in accuracy within each round, relative to round 5, the last round before field data were introduced. In all configurations, including μ=0, accuracy decreases when field data enter the federation in round 6, and by round 8 every configuration remains 1.4 to 2.2 points below its round 5 accuracy. The decline therefore occurs without any regularization and foes not grow with μ. FedAvg and the most strongly regularized configuration end at practically the same distance from their round 5 accuracy. Before the introduction of field data, no regularization related degradation is visible either: between rounds 4 and 5, the weakly regularized configurations lose 0.2 (μ=0) and 1.0 (μ=0.01) points. Because the local updates are small, the regularization does not prevent the clients from adapting to their local disease distributions. Consequently, the early-round advantage of FedProx over FedAvg at μ=0.01, where the proximal gradient is negligible, is more plausibly attributable to run-to-run variability than to the regularization itself.

Across all participating clients, the comparative analysis reveals clear and consistent distinctions in the behavior and robustness of the three algorithms considered. Overall, the results indicate that no single method dominates universally. This is a finding that is itself a meaningful contribution, since it demonstrates that algorithm selection in agriculturally heterogeneous FL deployments cannot be resolved by a one-size-fits-all default, as is common practice in much of the prior literature reviewed earlier. FedProx stands out as the most consistent performer across clients in absolute accuracy terms, but a closer reading of the per-client results exposes a pattern that a purely aggregate comparison would miss: FedProx accuracy tends to peak in the earliest rounds and drift gradually downward thereafter (observed in Clients 1, 3, and 6), suggesting that its proximal regularization, while effective at anchoring early local updates to the global model, may increasingly constrain adaptation as clients specialize toward their local, environmentally determined disease profiles over successive rounds. FedAvgM, conversely, proves advantageous specifically for clients where momentum can exploit a clearer, more consistent optimization signal. FedAvg remains competitive mainly in more balanced or less heterogeneous client scenarios, but lacks any explicit mechanism to counteract drift, leaving it vulnerable exactly where the environmentally grounded design introduces the greatest heterogeneity. Together, these findings support a central claim of this work: the choice of federated aggregation strategy should be informed by the specific agroecological profile of each client rather than fixed a priori, a conclusion that could not have been drawn from the synthetic, statistically partitioned non-IID benchmarks used in prior studies.

## 4. Conclusions

This work carried out a comparative analysis of three federated learning optimization strategies (FedAvg, FedProx, and FedAvgM) within a challenging, real world plant disease recognition task. All algorithms were implemented on a unified infrastructure, with consistent training protocols, identical backbone architectures, and controlled hyperparameters. This design allowed us to isolate the behavioral differences in each method and evaluate their effectiveness under realistic cross-client imbalance. The results show that FedAvgM stabilizes and improves performance, particularly for clients with more balanced local distributions. FedProx performs best for clients affected by strong data skew or limited local representativeness, confirming its robustness against client drift. FedAvg remains competitive on clients with simpler distributions but shows clear sensitivity to non-IID conditions.

This study makes several contributions to federated learning for plant disease recognition. First, we introduce an environmentally grounded FL framework in which client data distributions are derived from distinct climatic conditions rather than artificially generated statistical partitions. The resulting disease distributions are partially overlapping and locally incomplete, reflecting the statistical heterogeneity that naturally occurs across different agricultural environments. To our knowledge, this is the first study to evaluate FedAvg, FedProx, and FedAvgM under this type of agronomically motivated heterogeneity, rather than under synthetic non-IID benchmarks. Second, our framework aggregates only the shared feature extraction backbone across clients, while keeping the classifier heads fully local. This allows each client to maintain a classifier specialized to its own disease profile without sacrificing the benefits of collaborative representation learning. Third, we introduce a stabilizer node that preserves global knowledge across clients with partially overlapping and incomplete label distributions, addressing a scenario that has not been previously studied in the plant disease federated learning literature. Fourth, we analyzed the impact of training on real-world, imperfect data collected from the field, for clients who had previously trained their models exclusively on perfect data.

The experimental results also reveal the limitations of existing methods. No single aggregation algorithm performs optimally across all clients, since the problem is governed by many interacting factors that cannot be fully captured through simulation. When clients differ in the number of classes, the shared backbone and private head strategy is a promising start, since it makes the federation possible despite incompatible classifiers. Also, for short training horizons and simple classification tasks, FedAvg is sufficient, since FedProx behaves almost identically with a small proximal coefficient.

Future work will extend this analysis in several directions, including the incorporation of additional aggregation algorithms and adaptive personalization layers, as well as the exploration of larger backbone architectures and vision transformers under federated training.

## Figures and Tables

**Table 1:** Own dataset specifications.

Host	Class	Before Augmentation	After Augmentation
Tomato	Bacterial spot	374	2244
Tomato	Early blight	60	360
Tomato	Healthy	107	643
Tomato	Late blight	186	1116
Tomato	Septoria leaf	28	168

**Table 2:** Public dataset distribution for *client 1*.

Host	Class	Train	Valid
Tomato	Early_blight	2501	480
Tomato	Septoria_leaf	3952	436

**Table 3:** Private dataset distribution for *client 1*.

Host	Class	Train	Valid
Tomato	Early_blight	306	54
Tomato	Septoria_leaf	134	24

**Table 4:** Dataset distribution for *client 2*.

Host	Class	Train	Valid
Tomato	Bacterial_spot	3355	425
Tomato	Early_blight	2501	480
Tomato	Target_spot	3688	457

**Table 5:** Dataset distribution for *client 3*.

Host	Class	Train	Valid
Tomato	Leaf_mold	2834	470
Tomato	Mosaic_virus	2611	448

**Table 6:** Dataset distribution for *client 4*.

Host	Class	Train	Valid
Tomato	Bacterial spot	3355	425
Tomato	Early blight	2501	480
Tomato	Healthy	4461	481
Tomato	Late blight	4230	463
Tomato	Leaf mold	2834	470
Tomato	Mosaic virus	2611	448
Tomato	Septoria leaf spot	3952	436
Tomato	Target spot	3688	457
Tomato	Two-spotted spider mite	3852	435
Tomato	Yellow leaf curl virus	5659	490

**Table 7:** Dataset distribution for *client 5*.

Host	Class	Train	Valid
Tomato	Mosaic_virus	2611	448
Tomato	Two_spotted_spider_mite	3852	435
Tomato	Yellow_leaf_curl	5659	490

**Table 8:** Public dataset distribution for *client 6*.

Host	Class	Train	Valid
Tomato	Healthy	4461	481
Tomato	Late_blight	4230	463

**Table 9:** Private dataset distribution for *client 6*.

Host	Class	Train	Valid
Tomato	Healthy	547	96
Tomato	Late_blight	949	167

**Table 10:** Configuration of head and finetune parameters per client in the second scenario.

Client ID	Classes	Class No.	Head	Finetune
**Client 1**	Early blight, Septoria leaf	2	6	2
**Client 2**	Bacterial spot, Early blight, Target spot	3	8	3
**Client 3**	Leaf Mold, Mosaic virus	2	6	2
**Client 4**	Bacterial spot, Early blight, Healthy, Late blight, Leaf Mold, Mosaic virus, Septoria leaf, Target spot, Two spotted spider mite, Yellow Leaf Curl	10	12	4
**Client 5**	Mosaic virus, Two spotted spider mite, Yellow Leaf Curl	3	8	3
**Client 6**	Healthy, Late blight	2	6	2

**Table 11:** *Client 1*: per-round validation accuracy in Scenario 1 (5 rounds) under FedAvg, FedProx and FedAvgM.

Round	FedAvg (%)	FedProx (%)	FedAvgM (%)
1	0.7478	0.9192	0.7631
2	0.7533	0.9225	0.7675
3	0.8919	0.9225	0.7795
4	0.8079	0.9127	0.8876
5	0.7969	0.8963	0.8865

**Table 12:** *Client 1*: per-round validation accuracy in Scenario 2 (8 rounds) under FedAvg, FedProx and FedAvgM.

Round	FedAvg (%)	FedProx (%)	FedAvgM (%)
1	0.9301	0.9356	0.8952
2	0.9367	0.9389	0.9170
3	0.9356	0.9498	0.9301
4	0.9323	0.9476	0.9356
5	0.9432	0.9421	0.9378
6	0.8590	0.8333	0.8301
7	0.8625	0.8219	0.8366
8	0.8635	0.8268	0.8375

**Table 13:** *Client 2*: per-round validation accuracy in Scenario 1 (5 rounds) under FedAvg, FedProx and FedAvgM.

Round	FedAvg (%)	FedProx (%)	FedAvgM (%)
1	0.8010	0.7885	0.7577
2	0.7827	0.7930	0.8289
3	0.7952	0.7651	0.8774
4	0.7658	0.7372	0.9046
5	0.7599	0.7107	0.8950

**Table 14:** *Client 2*: per-round validation accuracy in Scenario 2 (8 rounds) under FedAvg, FedProx and FedAvgM.

Round	FedAvg (%)	FedProx (%)	FedAvgM (%)
1	0.7981	0.7981	0.7327
2	0.8811	0.8634	0.8840
3	0.8847	0.8649	0.8634
4	0.9170	0.8972	0.8781
5	0.8957	0.8510	0.8950
6	0.8744	0.8620	0.9258
7	0.8789	0.8847	0.9031
8	0.9325	0.8906	0.9273

**Table 15:** *Client 3*: per-round validation accuracy in Scenario 1 (5 rounds) under FedAvg, FedProx and FedAvgM.

Round	FedAvg (%)	FedProx (%)	FedAvgM (%)
1	0.7113	0.9041	0.9597
2	0.8420	0.9074	0.8976
3	0.7778	0.8595	0.9880
4	0.7723	0.8235	0.9706
5	0.6885	0.7789	0.9609

**Table 16:** *Client 3*: per-round validation accuracy in Scenario 2 (8 rounds) under FedAvg, FedProx and FedAvgM.

Round	FedAvg (%)	FedProx (%)	FedAvgM (%)
1	0.9325	0.9510	0.9183
2	0.9684	0.9837	0.9325
3	0.9793	0.9847	0.9597
4	0.9880	0.9858	0.9532
5	0.9826	0.9847	0.9739
6	0.9847	0.9826	0.9695
7	0.9891	0.9880	0.9826
8	0.9902	0.9901	0.9815

**Table 17:** *Client 4*: per-round validation accuracy in Scenario 2 (8 rounds) under FedAvg, FedProx and FedAvgM.

Round	FedAvg (%)	FedProx (%)	FedAvgM (%)
1	0.6209	0.6731	0.6236
2	0.7258	0.7514	0.6927
3	0.7442	0.7736	0.7442
4	0.7839	0.8129	0.7677
5	0.7900	0.7843	0.8096
6	0.7974	0.8399	0.8109
7	0.7924	0.8212	0.8430
8	0.7878	0.8425	0.8565

**Table 18:** *Client 5*: per-round validation accuracy in Scenario 1 (5 rounds) under FedAvg, FedProx and FedAvgM.

Round	FedAvg (%)	FedProx (%)	FedAvgM (%)
1	0.9126	0.9279	0.8813
2	0.9286	0.9126	0.9133
3	0.9272	0.8980	0.9155
4	0.9206	0.8951	0.9184
5	0.9235	0.9009	0.9133

**Table 19:** *Client 5*: per-round validation accuracy in Scenario 2 (8 rounds) under FedAvg, FedProx and FedAvgM.

Round	FedAvg (%)	FedProx (%)	FedAvgM (%)
1	0.9039	0.9439	0.9461
2	0.9301	0.9301	0.9337
3	0.9352	0.9483	0.9599
4	0.9425	0.9476	0.9250
5	0.9410	0.9483	0.9446
6	0.9374	0.9454	0.9534
7	0.9403	0.9592	0.9716
8	0.9352	0.9483	0.9774

**Table 20:** *Client 6*: per-round validation accuracy in Scenario 1 (5 rounds) under FedAvg, FedProx and FedAvgM.

Round	FedAvg (%)	FedProx (%)	FedAvgM (%)
1	0.5371	0.9301	0.5307
2	0.6515	0.9195	0.5286
3	0.6706	0.9078	0.5710
4	0.7479	0.8919	0.6642
5	0.6525	0.8824	0.7044

**Table 21:** *Client 6*: per-round validation accuracy in Scenario 2 (8 rounds) under FedAvg, FedProx and FedAvgM.

Round	FedAvg (%)	FedProx (%)	FedAvgM (%)
1	0.9195	0.9555	0.9502
2	0.9555	0.9661	0.9502
3	0.9756	0.9587	0.9608
4	0.9735	0.9449	0.9682
5	0.9725	0.9693	0.9703
6	0.9049	0.8973	0.8897
7	0.8707	0.8935	0.8973
8	0.8859	0.8939	0.8935

**Table 22:** FedProx regularization ablation (Scenario 2, mean over clients).

μ	Acc. R5	Final Acc. (R8)	$\Delta_{5\to 6}$	$\Delta_{5\to 8}$	Cross-Client Std	Grad. Ratio	Rel. Drift
0 (FedAvg)	0.921	0.899	−2.79	−2.17	0.070	–	–
0.01	0.913	0.899	−1.97	−1.44	0.062	–	–
0.1	0.846	0.828	−4.41	−1.85	0.058	$1.05\times 10^{-3}$	0.049
1.0	0.862	0.841	−2.63	−2.15	0.097	$5.79\times 10^{-3}$	0.019

## Data Availability

The real-field dataset generated and analyzed during the current study is not publicly available due to confidentiality considerations but is available from the corresponding author upon reasonable request.

## Abbreviations

The following abbreviations are used in this manuscript:

FL	Federated learning
FTL	Federated Transfer Learning
CNN	Convolutional neural network
ML	Machine learning
IID	Independent identically distributed
SGD	Stochastic Gradient Descent
FedAvg	Federated Averaging
FedProx	Federated Proximal
FedAvgM	Federated Averaging with Server Momentum
IoT	Internet of Things
